# Photochromic Molybdate for Advancing Anode Capacity of Lithium‐Ion Battery

**DOI:** 10.1002/advs.202519866

**Published:** 2025-11-30

**Authors:** Xiao‐Yue Zhang, Jian‐Ping Chen, Ping‐Wei Cai, Shou‐Tian Zheng, Cai Sun

**Affiliations:** ^1^ Fujian Provincial Key Laboratory of Advanced Inorganic Oxygenated‐Materials College of Chemistry Fuzhou University Fuzhou Fujian 350108 China; ^2^ Fujian Science & Technology Innovation Laboratory for Optoelectronic Information of China Fuzhou Fujian 350108 China

**Keywords:** electron transfer, Li‐ion battery, photochromism, polyoxometalate, polyoxomolybdate

## Abstract

Decoupling strategies can effectively enhance lithium‐ion battery (LIB) capacity, but suffer from the need for complex equipment, preventing widespread applicability. This work offers, for the first time, a decoupling strategy of electron‐transfer photochromism to improve LIB anode performance, without requiring complex equipment. The approach utilizes a new crystalline photochromic molybdate, MV[Mo_9_O_28_] (**1**, MV = methyl viologen cation) as the LIB anode. After UV irradiation, the initial **1** undergoes electron transfer from O to Mo, accompanied by a color change from colorless to blue and the generation of an ultra‐stable charge‐separated state with a lifetime of up to 2 years under ambient conditions. The observed capacity increases by 132 ± 14 mAh· g^−1^ across various current densities after coloration, along with superior rate performance—retaining 7.6% more capacity than the initial state even at a 100‐fold higher current density. Theoretical calculations confirm that the enhanced capacity and rate performance are attributable to the stable charge‐separated state. This unprecedented photochromic charge‐separated strategy contributes to the exploration of new outstanding anode materials for LIBs.

## Graphical abstract

Photochromic polyoxomolybdate is first used to enhance the anode capacity and rate performance of Li‐ion batteries.

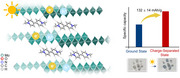

## 1 Introduction

The burgeoning global demand for advanced technologies, such as large‐scale energy storage systems, electric vehicles, and consumer electronics, has driven the need for rechargeable batteries that combine high safety, eco‐friendliness, cost‐effectiveness, and superior energy density. Lithium‐ion batteries (LIBs), known for their high energy density and long lifespan, have become widely utilized in portable electronic devices and electric vehicles.^[^ ^1^, ^2^, ^3^ ^]^ However, the limited theoretical capacity of commercial graphite anode (≈372 mAh g^−1^), and the sluggish kinetics of Li^+^ intercalation/deintercalation restrict their potential to meet the growing energy and power demands of next‐generation energy storage systems.^[^ ^4^, ^5^ ^]^ Exploring novel anode materials with high specific capacity and fast rate performance, to fulfil the primary requirements of LIBs, remains a long‐standing and challenging pursuit.

**Figure 1:**
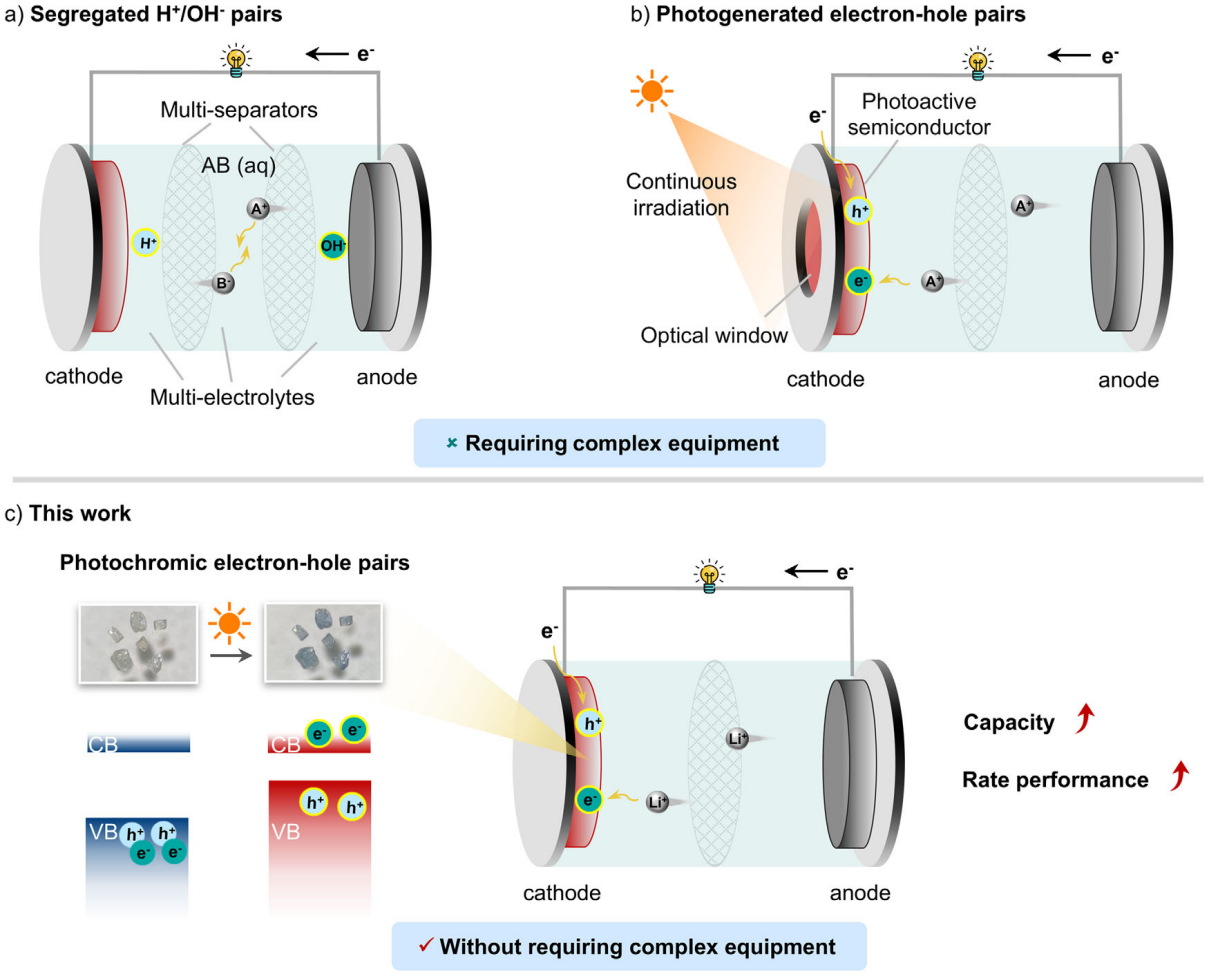
Decoupling strategy. a) Segregated H^+^/OH^−^ pairs generated by multi‐separators. b) Photogenerated electron–hole pairs requiring continuous irradiation. c) Photochromic electron–hole pairs without requiring complex equipment.

Numerous strategies have been reported to enhance the capacity of batteries, including electrolyte optimization,^[^ ^6^ ^]^ and electrode material modification.^[^ ^7^, ^8^ ^]^ In recent years, the decoupling strategy with additional H^+^/OH^−^ pairs or electron–hole pairs, offers an efficient approach to enhance battery performance (**Figure** **1**). For example, electrolyte decoupling enables the anode to work in an alkaline electrolyte and the cathode operates in an acidic electrolyte, separately, the H^+^/OH^−^ pairs participate in the cathode/anode reactions, respectively, contributing additional capacity to the system (Figure 1a).^[^ ^9^, ^10^, ^11^, ^12^ ^]^ Moreover, photoactive semiconductor electrodes can generate photogenerated electron–hole pairs under light illumination, and the efficient separation and transport of these photogenerated charge carriers can significantly enhance the battery capacity (Figure 1b).^[^ ^13^, ^14^, ^15^ ^]^ However, the former approach requires a physical separator, which increases the complexity and cost of the battery, while the latter depends on continuous light illumination to achieve capacity enhancement. The development of a decoupling method without complex equipment for enhancing the capacity of LIBs remains a challenge.

Electron‐transfer photochromic materials have been reported to form charge‐separated states after coloration,^[^ ^16^, ^17^ ^]^ in which electrons and holes remain stable for prolonged durations. It has been recently demonstrated that *N*‐heterocyclic aromatic cations, particularly methylviologen (MV), can further effectively stabilize these states through *π*‐cation polarization effects.^[^ ^18^ ^]^ Meanwhile, molybdenum oxides (MoO_x_) have been extensively investigated as anode materials for LIBs owing to their high theoretical capacity and environmental friendliness.^[^ ^19^, ^20^, ^21^, ^22^ ^]^ Inspired by these, we synthesized a novel inorganic–organic hybrid molybdate, MV[Mo_9_O_28_] (**1**), by incorporating MV as the counteraction. Compound **1** exhibits photochromic behavior via photoinduced electron transfer (PIET) from O to Mo upon UV irradiation (Figure 1c). Remarkably, the charge‐separated state persists for up to 2 years under ambient conditions. Interestingly, the specific capacity increases by 132 ± 14 mAh·g^−1^ after coloration across various current densities, accompanied by significantly enhanced rate capability, demonstrating 7.6% higher capacity retention compared to the initial state, even at a 100‐fold increase in current density. Distinct from previously reported photo‐assisted batteries, our proposal strategy requires only a short UV‐light stimulus to achieve sustained enhancement in capacity. Comprehensive experiment and density functional theory (DFT) calculations reveal that the additional electrons and holes contribute to increasing the electrical energy stored in LIBs, leading to the enhanced specific capacity, and the charge‐separated state effectively accelerates Li^+^ ions transfer, enabling excellent rate capacity. To our knowledge, this is the first successful application of photochromic charge‐separated to enhance capacity in LIBs.

## 2 Results and Discussion

Compound **1** was synthesized by a hydrothermal reaction of Na_2_MoO_4_·2H_2_O and MVCl_2_ at 160 °C for 72 h with pH adjusted to 1 using HCl (4 m). The crystallographic phase purity of **1** was systematically characterized by powder X‐ray diffraction (PXRD, Figure S1, Supporting Information), IR spectrum (Figure S2, Supporting Information), and elemental analyses (experimental details provided in the Supporting Information). Thermogravimetric analysis (Figure S3, Supporting Information) reveals that **1** exhibits excellent thermal stability below 350 °C.

**Figure 2:**
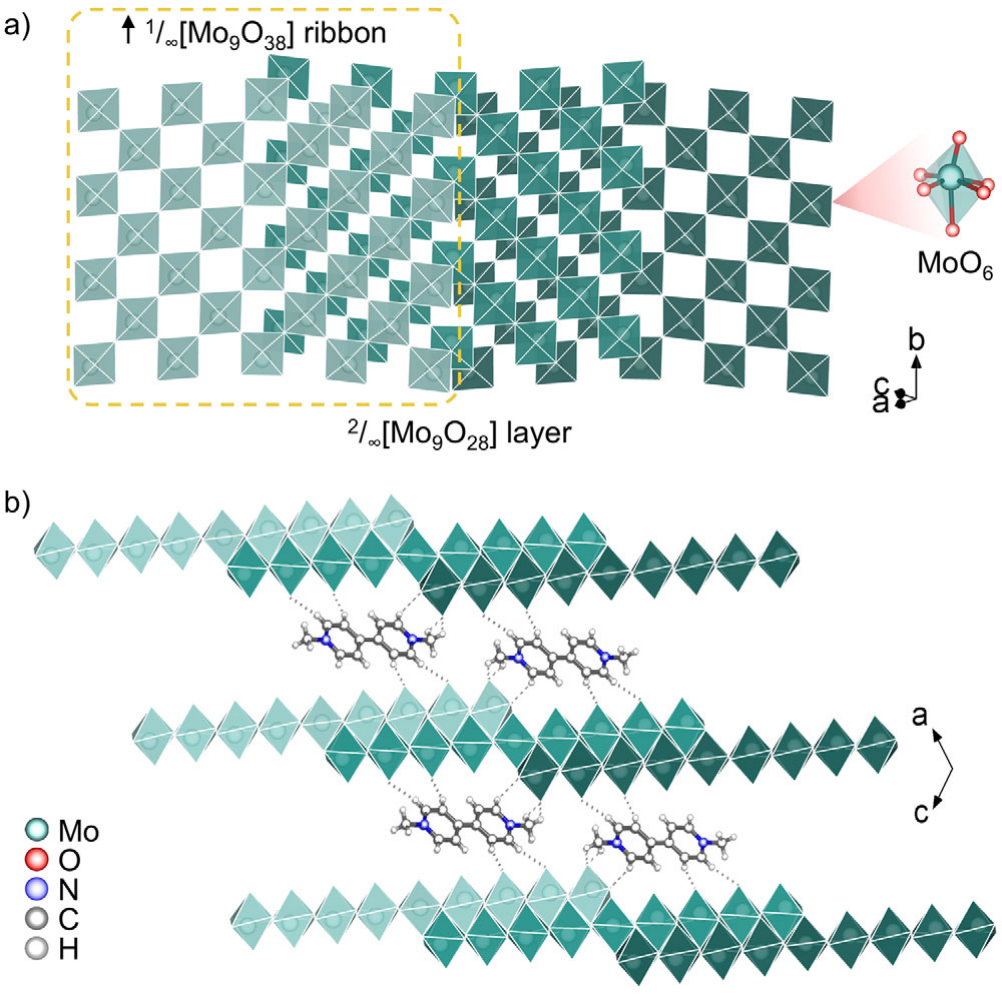
Structural of **1**. a) The stair‐like 2D ^2^/_∞_[Mo_9_O_28_]^2−^ layer. b) The pillared layered molybdate.

As shown in **Figures** **2** and S4 (Supporting Information), the crystal structure of **1** consists of 2D ^2^/_∞_[Mo_9_O_28_]^2−^ anionic layers separated by MV^2+^ cations. The layer belongs to the reported ^2^/_∞_[Mo_n_O_3n+1_]^2−^ family,^[^ ^23^ ^]^ with n = 9. The nine distorted [MoO_6_] octahedra connect each other by corner‐sharing in an interleaved manner to yield [Mo_9_O_46_] (Mo_9_) block unit. Then the Mo_9_ units further link themselves by corner‐sharing, resulting in an infinite ^1^/_∞_[Mo_9_O_38_] ribbon extending along the *b* direction. Each ribbon is sandwiched via edge‐sharing condensation between two adjacent ribbons shifted along in two opposite [101] directions with half‐overlapping, to form a stair‐like 2D ^2^/_∞_[Mo_9_O_28_]^2−^ layer, with the step width being exactly half of those of the ^1^/_∞_[Mo_9_O_38_] ribbon (Figure 2a). The interlayer spacing is 6.58 Å, with MV^2+^ inserted as counter‐cations between the layers, which may facilitate Li^+^ intercalation and diffusion, and establishes the structural basis for high Li^+^ storage capacity. The perpendicular distance from the N atom in MV^2+^ to the molecular plane of its adjacent MV^2+^ is 3.52 Å (Figure S5a, Supporting Information). A significant lateral offset exists between two adjacent MV^2+^ molecules, resulting in a distance of 4.76 Å from the N atom to the center of the pyridine of adjacent MV^2^^+^, indicating negligible *π*–*π* interactions. In addition, a wide hydrogen bonding network exists between the molybdate layer and MV^2+^ (Figure 2b; Figure S5b, Supporting Information), and the combination of strong electrostatic interactions and a diverse array of hydrogen bonding motifs contributes to the remarkable thermal stability of **1**.

**Figure 3:**
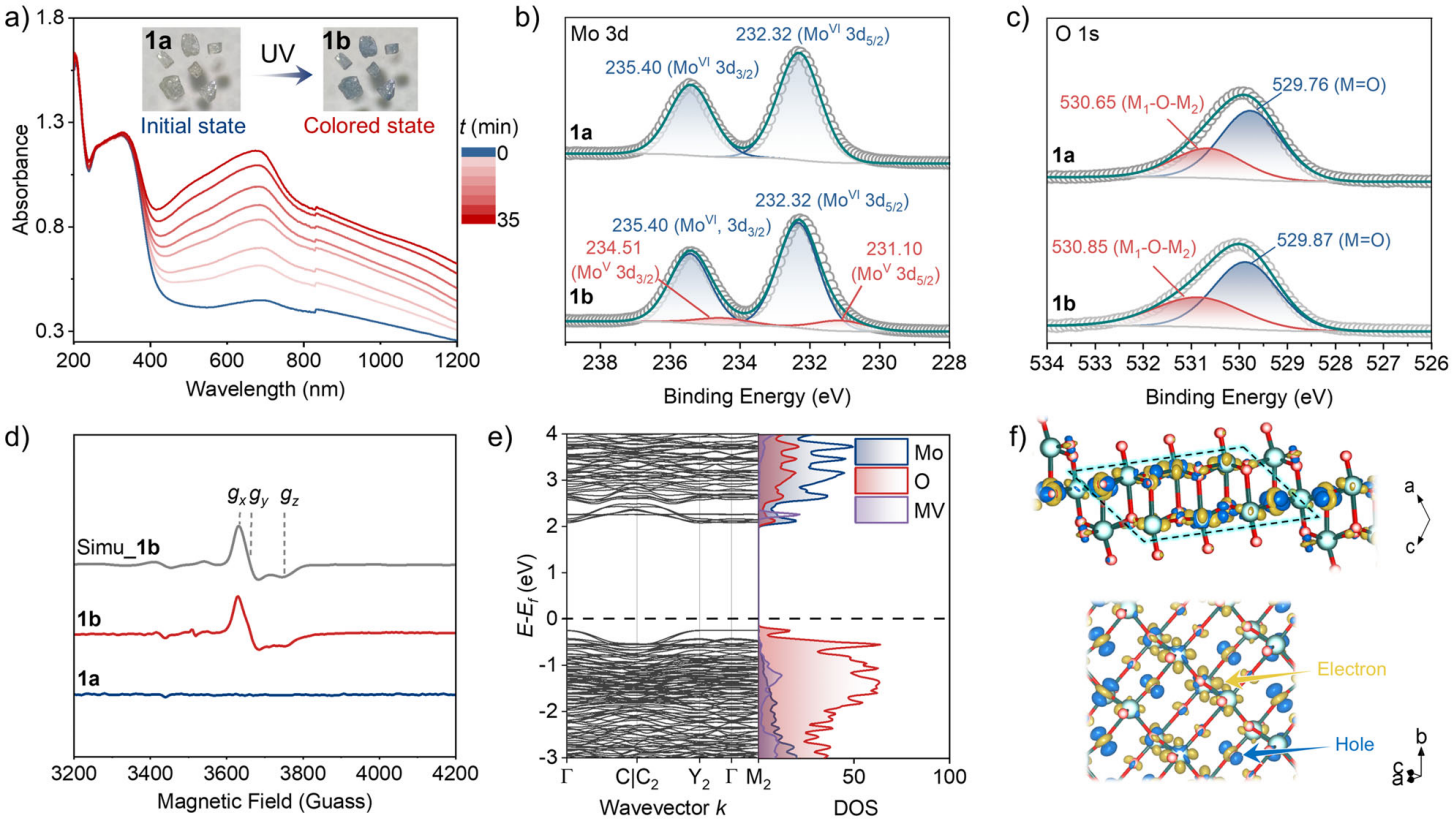
Photochromism of **1**. a) Time‐dependent UV–vis spectra, inset, Color change after UV irradiation. b‐c) XPS (Al‐Kα) core‐level spectra of Mo 3d and O 1s; d) EPR spectra of **1a**, **1b,** and simulated **1b**, where [*g*_x_, *g*_y_, *g*_z_] = [1.938, 1.921, 1.874], [*A*_x_, *A*_y_, *A*_z_] = [280, 380, 545] MHz. e) BS (left) and DOS (right). The Fermi level is set to zero. f) Electron density difference of molybdate layer, the dashed line indicates the primary charge‐separated region, yellow and blue colours represent charge accumulation and depletion after electron transfer, respectively, with an isosurface value of 0.004 e·Å^−3^.

Under ambient conditions, colorless crystal (**1a**) turns blue (**1b**) when irradiated with a 500 W Hg lamp (≈110 mW \to cm^−2^, default light source hereafter). Time‐dependent electron absorption spectra (**Figure** **3**a) show a new broad absorption band spanning 400–1200 nm for **1b**, with an absorption maximum centered at 681 nm. The coloration reaches saturation after 35 min of irradiation. To reveal the photochromic mechanism, comparative analysis of PXRD (Figure S1, Supporting Information) and IR (Figure S2, Supporting Information) data for **1a** and **1b** demonstrates no significant structural changes during the coloration process, confirming the absence of structural isomerization or phase transitions after irradiation. Next, as shown in Figure 3d, no electron paramagnetic resonance (EPR) signal is detected for **1a**, while characteristic unpaired electron signals are observed after coloration. The simulation of orthorhombic symmetry signals of *g*‐values at 1.938, 1.921, and 1.874, which are linked to superhyperfine coupling with the magnetic isotope Mo, conclusively demonstrates the presence of Mo^V^ and the occurrence of PIET. The orthorhombic symmetry features align with the structural characteristics of the distorted MoO_6_ octahedron. Therefore, the new absorption band can be attributed to an intervalence charge transfer (IVCT) process from Mo^V^ to Mo^VI^. The X‐ray photoelectron spectroscopy (XPS) data (Figure 3b,c; Figure S6, Supporting Information) provide further evidence for electron transfer behavior. Upon UV irradiation, significant changes are observed in the core‐level spectra of Mo 3d. Before coloration, there are two Mo 3d_3/2_ and 3d_5/2_ peaks of Mo^VI^ at ≈235.40 and 232.32 eV, respectively. After coloration, two new peaks emerge in the lower binding energy region of 234.51 and 231.10 eV, which can be attributed to the formation of partial Mo^V^. Further analysis of the O 1s spectral features reveals that the original peaks at 529.76 and 530.65 eV shift toward higher binding energies of 529.87 and 530.85 eV after coloration, indicating the O atoms as electron donors.

The band structure (BS) and densities of states (DOS) data reveal a bandgap of ≈2.5 eV (Figure 3e; Figures S7 and S8, Supporting Information). The valence band (VB) maximum is mainly dominated by O atoms, while the conduction band (CB) minimum is primarily contributed by Mo atoms, indicating the ability for O to Mo electronic transfer. Furthermore, the electron density difference (Δ*ρ*) isosurfaces (Figure 3f) demonstrate that electrons are transferred from O atoms to Mo atoms, leaving holes localized on O atoms, forming a charge‐separated state, and the electron transfer primarily occurs at the platform of the molybdate layer rather than at the step. This charge separation creates a favorable internal electric field on the platforms, which may accelerate Li^+^ transport. Surprisingly, the charge‐separated state remains stable even after two years under ambient conditions (Figure S9, Supporting Information). The observation is consistent with the strong π‐cation polarization effect of MV^2+^, which effectively stabilizes the charge‐separated state.^[^ ^18^ ^]^

**Figure 4:**
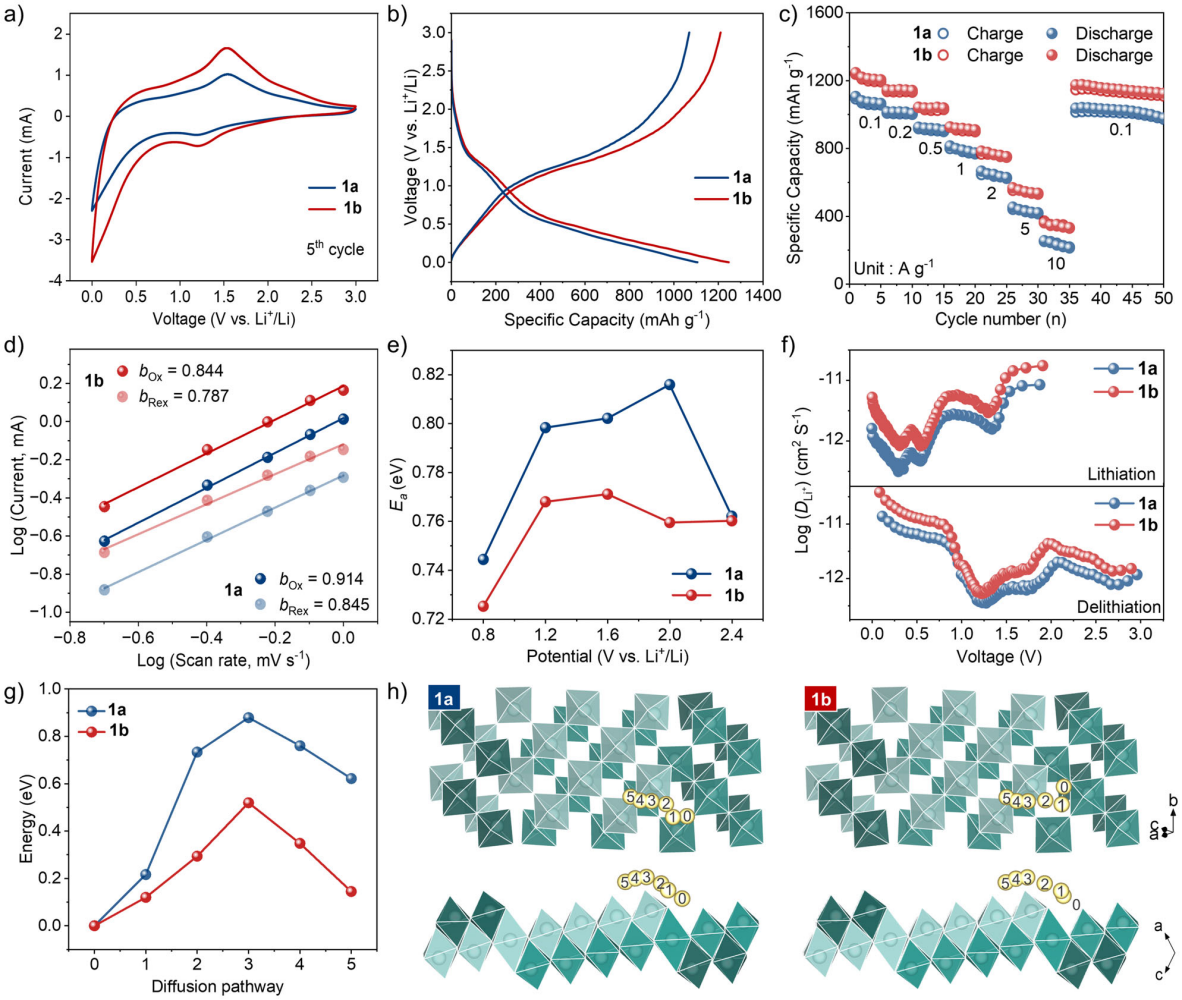
LIBs performance of **1**. a) The CV curves in the initial fifth cycle at 1.0 mV s^−1^; b) GCD curves at 1.0 mV s^−1^; c) Rate performance; d) Current response plotted against scan rates; e) Activation‐energy profiles at various potentials; f) Relative Li^+^ ions diffusivity at 0.05 A g^−1^; g,h) Simulated Li^+^ ions diffusion energy barrier, and diffusion pathways on the molybdate layer for **1a** and **1b**, respectively.

To investigate the impact of the charge‐separated state of **1** on LIB anode performance, we assembled 2032‐coin cells using **1** as anode materials and evaluated their performance. The cyclic voltammetry (CV) curves of **1a** and **1b** anodes (**Figure** **4**a; Figure S10, Supporting Information) show a reduction peak at 1.21 V, which can be attributed to the reduction of Mo^VI^ to Mo^IV^.^[^ ^21^, ^24^ ^]^ The nearly overlapped CV curves after the fifth cycle without significant changes demonstrate the reversibility of Li^+^ insertion/extraction into/from the anode. Moreover, the CV curves of **1b** exhibit a larger peak current density and integrated area compared with **1a**, indicating its higher capacity. The galvanostatic charge and discharge (GCD) curves (Figure S11, Supporting Information) at the first cycle deviate from subsequent ones due to the formation of the solid electrolyte interface (SEI) film. Comparing the second GCD profiles (Figure 4b) at a current density of 0.1 A g^−1^, the discharge specific capacities are 1098 mAh g^−1^ (61.44 Li^+^ ions) and 1245 mAh g^−1^ (69.65 Li^+^ ions) of **1a** and **1b**, respectively, which are consistent with the results calculated from the CV curves at 0.2 mV s^−1^ (Figure S12, Supporting Information), indicating that **1b** are conductive to promote the storage of a large amount of Li^+^ ions.

The rate capacities were further evaluated. As shown in Figure 4c, both electrodes **1a** and **1b** exhibit reversible discharge specific capacities; however, **1b** delivers consistently higher capacities by ≈132 ± 14 mAh g^−1^ than those of **1a** across various current densities. When the current density was increased by a factor of 100 (to 10 A g^−1^), the capacity retention rates for **1a** and **1b** were 21.5% and 29.1%, respectively, further confirming the superior rate performance of **1b**. When the current density returns to 0.1 A g^−1^, the capacity can be restored with a retention rate of 97.0%. The result can also be verified by the lower polarization voltage observed for the **1b** electrode (Figure S13, Supporting Information). Moreover, the specific capacity and rate performance of **1b** surpass those of excellent MoO_x_‐based anode materials (Table S4 and Figure S14, Supporting Information), further highlighting the significant advantages of the charge‐separated state in enhancing LIB capacity.

To investigate the origin of the capacity gain in the charge‐separated state, we further elucidate the charge storage mechanism of the LIB cells by collecting CV curves at different scan rates for electrodes **1a** and **1b** (Figures S15–S17, Supporting Information).^[^ ^19^ ^]^ As shown in Figure S18 (Supporting Information), the capacitive contribution percentage increases with scan rate for both electrodes. Notably, the capacitive contribution consistently dominates over the diffusion contribution across all scan rates, indicating capacitive‐dominated charge storage processes. Interestingly, after coloration, the proportion of diffusion contribution increases, suggesting that the charge‐separated state facilitates enhanced diffusion‐controlled faradaic contribution. The *b*‐values of the redox processes, which reflect the nature of the charge storage mechanism, are further analyzed. As displayed in Figure 4d, the *b*‐values for the redox processes are 0.914/0.845 and 0.884/0.787 for **1a** and **1b**, respectively, suggesting a charge storage mechanism leaning toward capacitive contributions. Specifically, the formation of the charge‐separated state induces an enhancement in the diffusive contribution, consistent with the discussion above.

The electrochemical impedance spectroscopy measurements are conducted to obtain the activation energy (*E_a_*) of the electrode reaction (Figures S19–S21, Supporting Information). The maximum *E*_a_ decreases from 0.82 eV in **1a** to 0.76 eV in **1b** (Figure 4e), indicating that electrode **1b** is more favorable than the **1a** thermodynamically, thereby facilitating lithiation/delithiation efficiency. Furthermore, galvanostatic intermittent titration technique tests (Figure S22, Supporting Information) are conducted to calculate the Li^+^ ion diffusion coefficients, demonstrating values of 3.83 × 10^−11^ to 5.36 × 10^−13^ cm^2^ S^−1^ for **1b** during lithiation/delithiation (Figure 4f). In contrast, **1a** exhibits significantly lower values of 8.79 × 10^−12^ to 3.14 × 10^−13^ cm^2^ S^−1^, highlighting the superior reaction kinetics of the **1b** electrode. The DFT simulations are further performed to calculate the diffusion barriers for Li^+^ ions on the surface of the molybdate layer. As shown in Figure 4g,h, the diffusion energy barrier for the charge‐separated state is substantially lower than that in the initial state. A lower diffusion barrier facilitates faster ion migration. Therefore, the charge‐separated state effectively enhances the interlayer Li^+^ migration rate, thereby further improving the overall charging and discharging kinetics, as well as the enhancement of the diffusive contribution for total capacity.

In order to gain insight into the charge storage mechanism, in situ XRD (Figure S23, Supporting Information) and ex situ XPS (Figure S24, Supporting Information) techniques are used to analyze the structure and valence state changes of **1b** during the charge/discharge process. The unchanged XRD patterns of **1b** indicate no new phase generation in the charge/discharge process. The core‐level Mo 3d spectra of electrode **1b** at open circuit voltage coincide with those in Figure 3b, exhibiting coexisting Mo^VI^ and Mo^V^ signatures. Upon discharging to 0.01 V, a new peak corresponding to Mo^IV^ emerges, confirming partial reduction of Mo atoms to obtain two‐electron during discharge. When recharged to 3.0 V, exclusively Mo^VI^ peaks are observed, demonstrating complete electron transfer from reduced Mo sites to the counter electrode via the external circuit.

**Figure 5:**
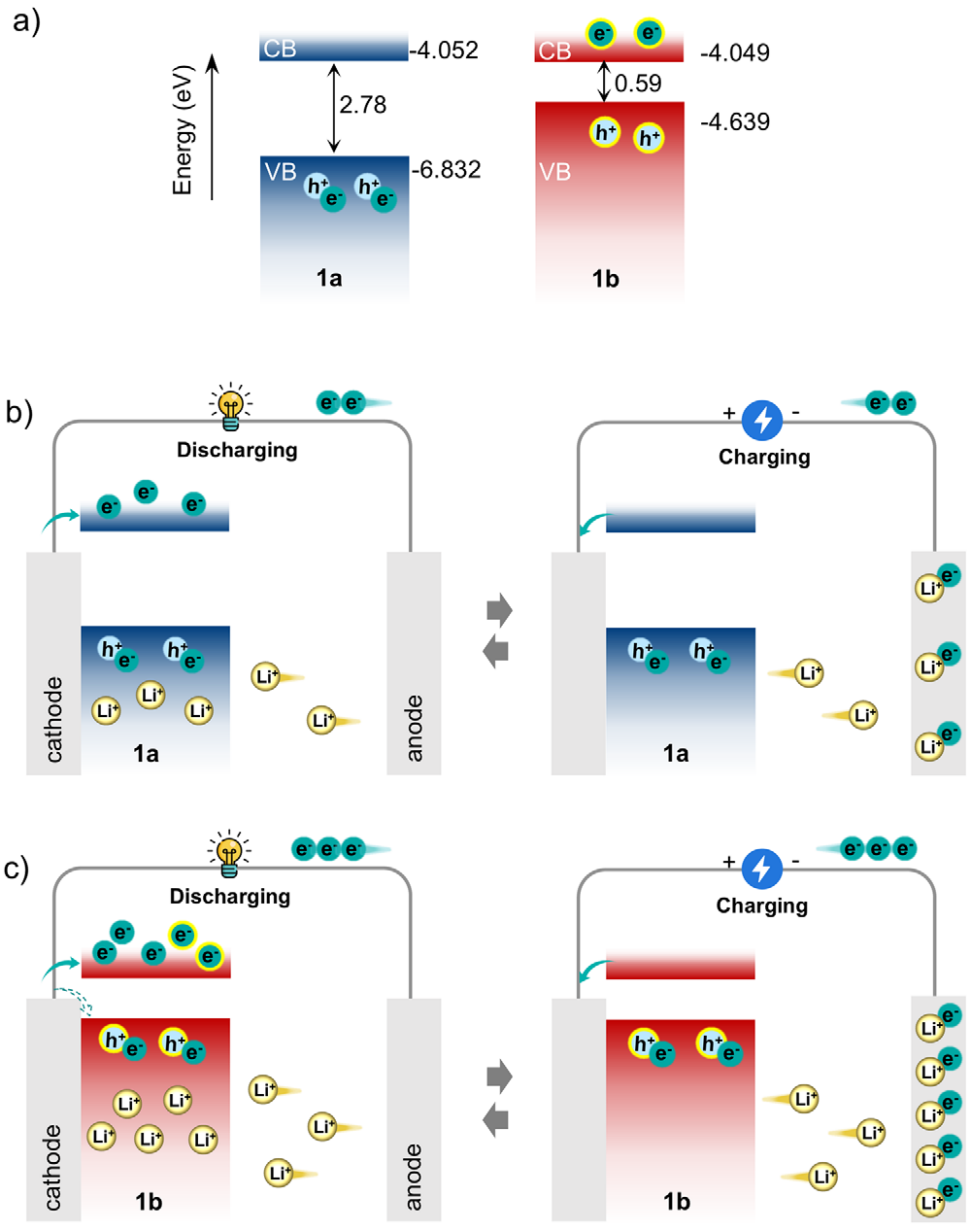
a) Schematic diagrams of band structure; b) Charging/discharging schematic diagrams showing advancing anode capacity of LIBs. Note, the electrons and holes of the charge‐separated state are outlined in yellow; the dashed arrow indicates partial recombination of additional electrons with holes in the VB during the first discharge cycle.

Based above results, a phenomenological explanation for the capacity gain after coloration is proposed, as schematically illustrated in **Figure** **5** and Video S1 (Supporting Information). The bandgaps and energy level alignments (relative to the vacuum level) of **1a** and **1b** (Figure 5a) are determined by UV–vis–NIR absorption spectra (Figure S25a,b, Supporting Information) and CV curves (Figure S25c,d, Supporting Information). For the **1a** electrode (Figure 5b), Li^+^ ions deintercalate from the anode and intercalate into **1a** during discharge, while electrons enter its CB via the external circuit. During charge, this process reverses—Li^+^ ions deintercalate from **1a** back to the anode, accompanied by oxidation of **1a** and electron extraction from its CB. When **1b** serves as the electrode (Figure 5c), it resides in a stable charge‐separated state with electrons in CB and holes in VB. During discharge, while electrons enter the CB of **1b** via the external circuit, partial recombination of additional electrons with holes occurs in the VB, thus enabling enhanced Li^+^ intercalation into the **1b** electrode. During charge, **1b** releases more electrons from its CB comparing with 1a, alongside greater Li^+^ deintercalation, thereby increasing charge capacity. The complete depletion of electrons from the CB of **1b** during charging corresponds to the disappearance of reduced Mo peaks observed in Figure S24 (Supporting Information). Crucially, the extra capacity gained during charging is reversibly released upon discharge. Since the additional holes in **1b** are consumed by recombination during the first cycle discharge, all subsequent discharge processes involve electrons entering the CB exclusively. This mechanism consistently sustains the high specific capacity of **1b** over extended cycling.

## 3 Conclusion

In summary, we report a novel decoupling strategy using electron‐transfer photochromism to enhance LIB anode performance without requiring complex equipment. After coloration, both capacity and rate performance are significantly improved, surpassing those of excellent MoO_x_‐based anode materials. The stable charge‐separated state not only provides additional electrons and holes, synergistically contributing to higher capacity, but also effectively accelerates Li^+^ ions transfer, enabling excellent rate capacity. This work presents the first example of enhanced LIB anode performance induced by the electron‐transfer photochromism, offering new insights into the strategy for developing high‐performance anode materials for LIBs.

## Conflict of Interest

The authors declare no conflict of interest.

## Supporting information



Supporting Information

Supplemental Video 1

Supporting Information

## Data Availability

The data that support the findings of this study are available in the supplementary material of this article.
