# Supplementary material for: Photochromic Molybdate for Advancing Anode Capacity of Lithium‐Ion Battery
Source: Adv Sci (Weinh). 2025 Nov 30;13(6):e19866. doi: 10.1002/advs.202519866 (PMC12866782; doi:10.1002/advs.202519866)
Supplement: Supplementary file 1 — Supporting Information [file ADVS-13-e19866-s002.pdf]

Supporting Information

Photochromic Molybdate for Advancing Anode Capacity of Lithium-Ion Battery

Xiao-Yue Zhang,[a]+ Jian-Ping Chen,[a]+ Ping-Wei Cai,[a] Shou-Tian Zheng,[a]* and Cai Sun[a], [b]**

[a]Fujian Provincial Key Laboratory of Advanced Inorganic Oxygenated-Materials, College of Chemistry, Fuzhou University, Fuzhou, Fujian 350108, China

[b]Fujian Science & Technology Innovation Laboratory for Optoelectronic Information of China, Fuzhou, Fujian 350108, China

E-mail: cai2022@fzu.edu.cn; stzheng@fzu.edu.cn; csun@fzu.edu.cn

[+] These authors contributed equally to this work.

Index

Experimental section 3

Supplementary Tables. 6

Table S1. Crystal and structure refinement data of **1** 6

Table S2. Selected bond lengths (Å) for **1**. 7

Table S3. Selected angles (°) for **1** 8

Table S4. Comparison of **1** with reported molybdate anode materials for LIBs. 10

Supplementary Figures. 14

Figure S1. PXRD patterns of **1**. 14

Figure S2. IR spectra of **1** 15

Figure S3. Thermogravimetric analysis of **1**. 16

Figure S4. Structural evolution of **1**. 17

Figure S5. Intermolecular interactions of **1**. 18

Figure S6. The XPS curves of Mo3d and O1s. 19

Figure S7. The band structure (BS) of **1** 20

Figure S8. The partial density of states (PDOS). 21

Figure S9. Stability test of charge-separated state of **1**. 22

Figure S10. CV curves at 1.0 mV s^{-1}. 23

Figure S11. The first five cycles galvanostatic charge-discharge curves. 24

Figure S12. CV comparison of **1a** and **1b** at a scan rate of 0.2 mV s^{-1}. 25

Figure S13. Charge-discharge curves. 26

Figure S14. Comparison of specific capacities with reported literature 27

Figure S15. CV curves at different scan rates. 28
Figure S16. Calculated total current and capacitive current of **1a**. 29
Figure S17. Calculated total current and capacitive current of **1b**. 30
Figure S18. Column diagram of the capacitive ratio at various scan rates 31
Figure S19. Nyquist plots of **1a**. 32
Figure S20. Nyquist plots of **1b**. 33
Figure S21. The equivalent circuit. 34
Figure S22. GITT curves. 35
Figure S23. *In-situ* XRD patterns of **1b**. 36
Figure S24. Mo 3d XPS spectra of **1b**. 37
Figure S25. Energy levels of **1**. 38
References 39

Experimental section

Materials

All chemicals were used as purchased without further purification.

Measurements

Elemental analyses of C, H, and N were measured on an Elementar Vario EL III microanalyzer. Infrared (IR) spectra (KBr pellet) were performed on an Opus Vetex 70 FT-IR infrared spectrophotometer in the range of 400–4000 cm^{-1}. ICP analyses were conducted on a Shimadzu ICPE-9820 (ICP-OES). UV-Vis-NIR spectra were performed on a SHIMADZU UV-2600 and SHIMADZU UV-3600i plus by using the $BaSO_4$ as the blank. Powder X-ray diffraction (PXRD) patterns were recorded on a Rigaku MiniFlex 600 diffractometer with CuK_α radiation (λ = 1.54056 Å). Simulated PXRD pattern was derived from the Mercury Version 4.3.0 software using the X-ray single crystal diffraction data. Thermogravimetric analyses were conducted using a Mettler Toledo TGA/DSC 3+ analyzer in an N_2-flow atmosphere with a heating rate of 10 °C/min at a temperature of 30–800 °C. Electron paramagnetic resonance (EPR) spectra were recorded on a Bruker ER-420 spectrometer with a 100 kHz magnetic field in the X band at room temperature (RT). X-ray photoelectron spectroscopy (XPS) studies were performed in a ThermoFisher ESCALAB250 X-ray photoelectron spectrometer (powered at 150 W) using AlK_α radiation (λ = 8.357 Å).

Synthesis of MV[Mo_9O_{28}] (1, MV = methyl viologen cation)

Na_2MoO_4 $2H_2O$ (151 mg, 0.625 mmol) and $MVCl_2$ (65 mg, 0.252 mmol) in 8 mL water were stirred at room temperature for 5 minutes to yield a clear liquid. The pH was adjusted to 1 with 4 M HCl, and the solution becomes cloudy immediately. After stirring at room temperature for 1 h, the mixture was put into a 25 mL Teflon-lined stainless steel autoclave at 140 °C for 72 h. In most cases, the yielded crystals were colorless block crystals of **1** (ca. 34.5% yield based on MV). Elemental analysis: Found: C, 9.80; H, 1.08; N, 1.85%, Mo, 57.14%; Calc. C, 9.62; H, 0.94; N, 1.87%, Mo, 57.65%. FT-IR (KBr, 4000–400 cm^{-1}) 3122 (w), 3050 (m), 1639 (s), 1562 (w), 1503 (w), 1442 (m), 1341 (w), 1268 (w), 1228 (w), 1182 (w), 921 (m), 898 (w), 867 (w), 826 (s), 742 (s), 695 (s), 641 (m), 539 (s), 456 (s) (Figure S2).

X-ray crystallography

Single-crystal X-ray diffraction measurements were performed on Rigaku XtaLAB Synergy-R, using graphite monochromated Mo K_α radiation (λ = 0.71073 Å). Intensity data sets were collected using ω scan techniques and corrected for *Lp* effects. The structures were solved by the direct method and refined by full-matrix least squares on F^2 using the Siemens SHELXTL^{TM} Version 5 package of crystallographic software with anisotropic thermal parameters for all non-hydrogen atoms. Hydrogen atoms were added geometrically and refined using the riding model. Crystal data and structure refinement results for **1** are summarized in Table S1.

The entries of CCDC-2464598 contain the supplementary crystallographic data for **1**. These data can be obtained free of charge at http://www.ccdc.cam.ac.uk/conts/retrieving.html or from the Cambridge Crystallographic Data Centre, 12, Union Road, Cambridge CB2 1EZ, U.K. Fax: (Internet) +44-1223/336-033. E-mail: depos-it@ccdc.cam.ac.uk.

Electrochemical Characterization

Anode preparation. The anode was fabricated by mixing active materials (70 wt%), acetylene black (20 wt%), and sodium carboxymethyl cellulose (10 wt%) into a uniform slurry, and followed to coated onto a copper foil. The pellets were dried in a vacuum at 60 °C for 24 h. The mass loading of the anode active material on each electrode (diameter: 12 mm) was ~1.2 mg cm^{-2}.

Electrochemical measurements. The electrochemical measurements were investigated with 2032-type coin cells assembled in a glove box filled with argon atmosphere (H_2O < 0.5 ppm, O_2 < 0.5 ppm). For LIBs, the Li metal foils as the counter/reference electrode, 1 M $LiPF_6$ dissolved in diethyl carbonate (DEC)/ethylene carbonate (EC)/methyl ethyl carbonate (MEC) (2:3:5 by wt%) as electrolyte and PP membrane as the separator. Then, the assembled cells were aged for 20 h to ensure that the system was fully wetted. The cell cycling experiments are carried out in a climatic chamber with a constant temperature of 35 °C. The electrochemical performances of the batteries were tested on a Land battery testing system (LANDTE Co., China) with the potential window of 0.01–3 V for LIBs, and CV tests were measured on a CHI660E electrochemical station (CHI Instrument Co., Shanghai, China), respectively. For the long-term cycling performance at high specific currents, the LIBs were first activated at 0.1 A g^{-1} for ten cycles and then were operated at a higher specific current for a long cycle test. An ac voltage amplitude of open-circuit voltage was employed to measure EIS within the frequency range from 0.01 Hz to 100 kHz. The specific capacity and specific currents were calculated based on the weight of anode materials. The galvanostatic intermittent titration technique (GITT) tests were measured under constant-current conditions at 0.05 A g^{-1} for 20 min and rest intervals for 40 min after the 10th cycles.

Activation energy of redox reaction. The LIBs were assembled as described above and were initially discharged and charged at 1 A g^{-1} for 5 cycles prior to the EIS tests. Subsequently, the cells were placed in a thermostatic chamber set to specific temperatures

(273, 283, 293, 303, 313, and 323 K) for 1 hour. Impedance spectra were then measured at various potentials ranging from 0.2 V to 2.8 V using the same electrode under the specified temperature. Finally, the activation energy of the redox reaction was calculated at different potentials.

Calculation of *b*-value. The electrochemical kinetics within the cell were investigated using cyclic voltammetry (CV) at various scan rates. The measured current from the CV curves can be attributed to two distinct charge-storage mechanisms: the diffusion-controlled faradaic contribution and the surface capacitive dominated processes. Generally, the relationship between peak current (i) and scan rate (v) can be described using the following formulas:[1]

$$i = a\, v^{b}$$

$$log(i) = b\, log(v) + log(a)$$

where a and b are the adjustable parameters. In the context of the redox process of the electrode, the b-value indicates the nature of the charge storage mechanism. Specifically, when b approaches 1.0, it suggests an ideally capacitive contribution process. On the other hand, when b approaches 0.5, it indicates a predominantly faradaic intercalation process.

Calculation of capacitive contribution:

The ratios of Li^{+} capacitive contribution can be further quantitatively distinguished by separating the current response (i) at a specific potential (V) according to the following equations:[2]

$$i(V) = k_1 v + k_2 v^{1/2}$$

$$i(V)/v^{1/2} = k_1 v^{1/2} + k_2$$

where both k_1 and k_2 are constant values obtained from the slope and intercept of the $i(V)/v^{1/2}$ versus $v^{1/2}$ plot, respectively. And $k_1 v$ and $k_2 v_{1/2}$ represent the capacitive contribution and diffusion contribution, respectively.

Calculation of D_{Li^+} value:

The Li ions diffusion coefficient (D_{Li^+}) can be calculated from the GITT potential profiles according to the simplified Fick's second law with the following equation:[3]

$$D = \frac{4}{\pi\tau}\left(\frac{m_B V_M}{M_B S}\right)^2\left(\frac{\Delta E_s}{\Delta E_\tau}\right)^2$$

where m_B is the electrode active mass and S (cm^2) is the geometric area. ΔE_s is the quasi-thermodynamic equilibrium potential difference between before and after the current pulse. ΔE_τ is the duration of the current pulse and the change of voltage during the current pulse. M_B (g mol^{-1}) is the molecular weight and V_M (cm^3 mol^{-1}) is molar volume of electrode material.

Calculation of activation energy:

The activation energy (E_a) was calculated using Arrhenius equation R_{ct} = $Ae^{-Ea/RT}$, where the reciprocal of the charge transfer resistance (R_{ct}) is linearly related to the reciprocal of the absolute temperature ($1/T$). The corresponding R_{ct} values of the electrode can be obtained by electrochemical impedance spectroscopy (EIS) measurements. The E_a values at various voltages are determined from the slopes obtained by fitting the charge-transfer impedances measured at different temperatures.

Computational approaches

Calculation of energy band structure and partial density of states

DFT calculations of energy band structure and partial density of states, were performed by using the Vienna ab initio Simulation Package (VASP).[4] The Kohn–Sham wave functions were expanded in a plane wave basis set with a cutoff energy of 400 eV and a 1 ×5 × 1 Monkhorst-Pack grid of *k*-point. The projector-augmented wave (PAW) method and PBE potential for the exchange correlation functional were used. All atoms were allowed to relax until the forces fell below 0.01 eV Å^{-1}.[5]

Calculation of electron density difference

The model of molybdate layer was taken from the single-crystal structure of **1**. The electron density before and after coloration have been simulated by using the CP2K software[6] at PBE function with basis set and pseudopotential being DZVP-MOLOPT-SR-GTH. Only Γ point has been considered for Brillouin zone sampling in the reciprocal space. The analysis of wave function for electron density difference was derived using the VESTA package.[7] Electron density difference calculations were conducted on the basis of the following equation: $\Delta\rho = \rho_{\text{colored state}} - \rho_{\text{initial state}}$, where $\rho_{\text{initial state}}$ and $\rho_{\text{colored state}}$ were electron densities of molybdate layer before and after irradiation, respectively.

Calculation of Li^{+} ions diffusion energy barrier and pathways

The integration over the reciprocal space was performed using a Monkhorst model with a Γ-point. The migration behavior of Li in different regions of the model was evaluated using the climbing-image NEB method.[8]

Supplementary Tables.

Table S1. Crystal and structure refinement data of **1**.

	**1**
Formula	$C_{12}H_{14}N_2O_{28}Mo_9$
Mr	1497.71
Crystal size (mm³)	0.12× 0.15 × 0.16
Crystal system	monoclinic
Space group	*C2/c*
a (Å)	26.8624(16)
b (Å)	5.4839(2)
c (Å)	24.6339(6)
α *(deg)*	90
β *(deg)*	122.636(6)
γ *(deg)*	90
V (Å³)	3055.9(3)
D_{calcd} *(g/cm³)*	3.255
Z	4
F(000)	2808.0
Abs coeff (mm⁻¹)	3.678
Refund collcd/unique (R_{int})	10974/2678 (0.0309)
Data/params/restraints	2678/0/240
R_1^a	0.0400
ωR_2^b	0.0896
GOF on F^2	1.083
$\Delta\rho_{max}$ *and* $\Delta\rho_{min}$ *(e/Å³)*	2.83 and -0.97

$^aR_1 = \sum||F_o|-|F_c||/\sum|F_o|$, $^b\omega R_2 = \{\sum\omega[(F_o)^2-(F_c)^2]^2/\sum\omega[(F_o)_2]^2\}^{1/2}$.

Table S2. Selected bond lengths (Å) for **1**.

Bond	Dist	Bond	Dist
Mo3-O4[1]	2.255(4)	Mo5-O13[4]	1.922(4)
Mo3-O10	1.904(3)	Mo5-O14[4]	1.724(4)
Mo3-O7	1.968(4)	Mo5-O14	1.724(4)
Mo3-O6[2]	2.333(4)	Mo5-O1[1]	2.248(4)
Mo3-O8	1.690(4)	Mo5-O1[5]	2.248(4)
Mo3-O9	1.701(4)	Mo4-O10	2.012(4)
Mo2-O4	1.914(4)	Mo4-O13	1.897(4)
Mo2-O7[1]	2.287(4)	Mo4-O1[1]	2.249(4)
Mo2-O7	1.950(4)	Mo4-O11	1.701(4)
Mo2-O6	1.720(4)	Mo4-O12	1.685(4)
Mo2-O3[2]	2.287(4)	Mo4-O9[2]	2.336(4)
Mo2-O5	1.694(4)	N1-C1	1.331(8)
Mo1-O4	2.022(3)	N1-C5	1.339(8)
Mo1-O10[1]	2.274(4)	N1-C6	1.470(9)
Mo1-O14[3]	2.340(4)	C1-C2	1.378(10)
Mo1-O3	1.733(4)	C2-C3	1.397(9)
Mo1-O1	1.828(4)	C4-C5	1.363(10)
Mo1-O2	1.699(4)	C4-C3	1.387(9)
Mo5-O13	1.922(4)	C3-C3[6]	1.472(13)

[1]3/2-X,1/2-Y,1-Z; [2]+X,1+Y,+Z; [3]3/2-X,3/2-Y,1-Z; [4]1-X,+Y,1/2-Z; [5]-1/2+X,1/2-Y,-1/2+Z; [6]2-X,1-Y,1-Z

Table S3. Selected angles (°) for **1**.

**Angle**	**(°)**	**Angle**	**(°)**
O4^{1}-Mo3-O6^{2}	78.54(13)	O14-Mo5-O13	102.47(17)
O10-Mo3-O4^{1}	74.09(14)	O14^{4}-Mo5-O13^{4}	102.47(17)
O10-Mo3-O7	143.31(17)	O14-Mo5-O13^{4}	96.75(17)
O10-Mo3-O6^{2}	78.26(15)	O14-Mo5-O14^{4}	105.0(2)
O7-Mo3-O4^{1}	73.44(14)	O14^{4}-Mo5-O1^{1}	164.67(18)
O7-Mo3-O6^{2}	78.99(14)	O14-Mo5-O1^{1}	88.79(14)
O8-Mo3-O4^{1}	164.57(18)	O14^{4}-Mo5-O1^{5}	88.79(14)
O8-Mo3-O10	103.95(18)	O14-Mo5-O1^{5}	164.67(18)
O8-Mo3-O7	102.78(18)	O1^{5}-Mo5-O1^{1}	78.47(19)
O8-Mo3-O6^{2}	86.07(17)	O10-Mo4-O1^{1}	72.90(14)
O8-Mo3-O9	102.9(2)	O10-Mo4-O9^{2}	77.96(14)
O9-Mo3-O4^{1}	92.51(16)	O13-Mo4-O10	143.70(17)
O9-Mo3-O10	100.27(17)	O13-Mo4-O1^{1}	73.72(15)
O9-Mo3-O7	97.77(17)	O13-Mo4-O9^{2}	80.84(15)
O9-Mo3-O6^{2}	171.01(17)	O1^{1}-Mo4-O9^{2}	76.59(13)
O4-Mo2-O7	143.06(17)	O11-Mo4-O10	101.31(18)
O4-Mo2-O7^{1}	73.66(14)	O11-Mo4-O13	104.43(18)
O4-Mo2-O3^{2}	80.85(15)	O11-Mo4-O1^{1}	158.32(18)
O7-Mo2-O7^{1}	72.58(17)	O11-Mo4-O9^{2}	81.78(18)
O7^{1}-Mo2-O3^{2}	78.67(13)	O12-Mo4-O10	97.06(18)
O7-Mo2-O3^{2}	78.29(15)	O12-Mo4-O13	101.86(19)
O6-Mo2-O4	98.26(17)	O12-Mo4-O1^{1}	99.03(17)
O6-Mo2-O7^{1}	94.43(16)	O12-Mo4-O11	102.4(2)
O6-Mo2-O7	98.83(17)	O12-Mo4-O9^{2}	174.11(19)
O6-Mo2-O3^{2}	173.03(17)	Mo2-O4-Mo3^{1}	107.99(15)
O5-Mo2-O4	104.28(18)	Mo2-O4-Mo1	143.6(2)
O5-Mo2-O7	103.80(17)	Mo1-O4-Mo3^{1}	105.39(16)
O5-Mo2-O7^{1}	163.47(17)	Mo3-O10-Mo1^{1}	108.87(17)
O5-Mo2-O6	102.08(19)	Mo3-O10-Mo4	144.1(2)
O5-Mo2-O3^{2}	84.81(17)	Mo4-O10-Mo1^{1}	102.06(14)
O4-Mo1-O10^{1}	71.60(14)	Mo4-O13-Mo5	118.9(2)
O4-Mo1-O14^{3}	77.52(15)	Mo3-O7-Mo2^{1}	104.90(15)
O10^{1}-Mo1-O14^{3}	76.39(13)	Mo2-O7-Mo3	144.0(2)
O3-Mo1-O4	96.40(16)	Mo2-O7-Mo2^{1}	107.43(17)
O3-Mo1-O10^{1}	94.40(16)	Mo2-O6-Mo3^{6}	173.8(2)
O3-Mo1-O14^{3}	170.15(17)	Mo5-O14-Mo1^{3}	168.2(2)
O3-Mo1-O1	101.38(18)	Mo1-O3-Mo2^{6}	172.6(2)
O1-Mo1-O4	143.64(18)	Mo1-O1-Mo5^{1}	152.1(2)
O1-Mo1-O10^{1}	75.60(15)	Mo1-O1-Mo4^{1}	109.43(17)
O1-Mo1-O14^{3}	79.95(14)	Mo5^{1}-O1-Mo4^{1}	94.01(14)
O2-Mo1-O4	99.41(17)	Mo3-O9-Mo4^{6}	169.2(2)
O2-Mo1-O10^{1}	161.30(17)	C1-N1-C5	120.0(6)
O2-Mo1-O14^{3}	85.74(17)	C1-N1-C6	121.1(6)
O2-Mo1-O3	103.0(2)	C5-N1-C6	118.9(6)
O2-Mo1-O1	106.99(19)	N1-C1-C2	120.6(6)

Angle	(°)	Angle	(°)
O13-Mo5-O13^{4}	148.2(2)	C1-C2-C3	121.0(6)
O13^{4}-Mo5-O1^{1}	82.15(15)	C5-C4-C3	121.0(6)
O13^{4}-Mo5-O1^{5}	73.29(15)	N1-C5-C4	121.4(6)
O13-Mo5-O1^{1}	73.29(14)	C2-C3-C3^{7}	122.5(7)
O13-Mo5-O1^{5}	82.15(15)	C4-C3-C2	116.0(6)
O14^{4}-Mo5-O13	96.74(17)	C4-C3-C3^{7}	121.5(7)

^{1}3/2-X,1/2-Y,1-Z; ^{2}+X,1+Y,+Z; ^{3}3/2-X,3/2-Y,1-Z; ^{4}1-X,+Y,1/2-Z; ^{5}-1/2+X,1/2-Y,-1/2+Z; ^{6}+X,-1+Y,+Z; ^{7}2-X,1-Y,1-Z

Table S4. Comparison of **1** with reported molybdate anode materials for LIBs.

Electrode materials	Current density (A g^{-1})	Reversible capacity (mAh g^{-1})	Ref.
**1b**	0.1	1206	This work
	0.2	1146	
	0.5	1043	
	1	920	
	2	765	
	5	547	
	10	353	
**1a**	0.1	1068	
	0.2	1018	
	0.5	921	
	1	793	
	2	647	
	5	432	
	10	232	
PMo_{12}/CoTPyP	0.125	1486	[9]
	0.25	1163	
	0.5	941	
	1	719	
	2	536	
	4	372	
	8	223	
MoO_2/C	0.2	880	[10]
	0.5	700	
	1	600	
	2	510	
	3	460	
	4	440	
$PMo_{10}V_2$/PDA	0.02	995.7	[11]
	0.05	968.5	
	0.1	933.3	
	0.2	881.6	
	0.4	796.1	
	0.5	743.6	
	0.8	708.8	
	1	677.9	
	1.5	627.6	
	2	559.6	
MIL-88A@PMo_{12}	0.1	882	[12]
	0.2	820	
	0.4	750	
	0.8	650	
	1	610	
	2	490.8	
Co_3O_4/$CoMoO_4$	0.1	797.2	[13]
	0.2	686.4	
	0.5	465.9	
	1	301.7	

Electrode materials	Current density (A g^{-1})	Reversible capacity (mAh g^{-1})	Ref.
	2	166.8	
CuP_6Mo_{18}@ZnO/CNFs	0.1	758.2	[14]
	0.1	758.2	
	0.2	627.5	
	0.5	562.7	
	1	512.7	
	2	465.4	
SiO_2-PMo_{12}@rGO	0.1	720	[15]
	0.2	699	
	0.5	505	
	1	377	
	2	293	
Cu-POMOF@PPy	0.1	583.94	[16]
	0.2	463.94	
	0.4	399.83	
	0.5	381.33	
	1	298.83	
	2	202.17	
	4	133.27	
	5	115.25	
PMo_{12}@PPy/$Ti_3C_2T_x$	0.05	400	[17]
	0.1	350	
	0.2	300	
	0.5	220	
	1	180	
	2	130	
CuP_6Mo_{18}	0.1	396.8	[14]
	0.2	260.8	
	0.5	183.3	
	1	134.2	
	2	79.5	
PMo_{12}–PPy/RGO	0.1	1057.5	[18]
	0.2	905.9	
	0.5	688	
	1	510.7	
	2	316.4	
MZC-PMA-0.036	0.05	1311	[19]
	0.1	1026	
	0.2	905	
	0.5	751	
	1.0	615	
	2.0	474	
NENU-507	0.05	1024	[20]
	0.1	868	
	0.2	767	
	0.5	480	
PBG@PMo_{12}	0.1	669.9	[21]
	0.2	580	

Electrode materials	Current density (A g^{-1})	Reversible capacity (mAh g^{-1})	Ref.
	0.5	420	
	1.0	330	
	2.0	280	
	2.0	320	
α-MoO_3/η-Mo_4O_{11}	0.1	350	[22]
	0.2	300	
	0.5	265	
	1.0	230	
	2.0	190	
	5.0	140	
α-MoO_3	0.1	280	
	0.2	225	
	0.5	175	
	1.0	140	
	2.0	110	
	5.0	80	
η-Mo_4O_{11}	0.1	330	
	0.2	260	
	0.5	210	
	1.0	140	
	2.0	80	
	5.0	50	
MoO_2-Mo_2C@CNFs	0.2	697	[23]
	0.4	651	
	0.6	639	
	0.8	610	
	1.0	588	
	2.0	551	
	5.0	328	
	10.0	161	
MoO_2@C nanoflowers	0.1	188	[24]
	0.2	150	
	0.5	124	
	1.0	108	
	2.0	92	
	4.0	80	
	6.0	71	
	8.0	67	
MoO_2@C nanoparticles	0.1	123	
	0.2	107	
	0.5	88	
	1.0	74	
	2.0	63	
	4.0	46	
	6.0	37	
	8.0	36	
NC-MoO_3-400	0.1	1200	[25]
	0.3	860	
	0.5	780	

Electrode materials	Current density (A g^{-1})	Reversible capacity (mAh g^{-1})	Ref.
	1.0	620	
	2.0	480	
	5.0	450	
	10.0	400	
N-MoO_3-500	0.1	700	
	0.3	480	
	0.5	410	
	1.0	360	
	2.0	210	
	5.0	170	
	10.0	80	

Supplementary Figures.

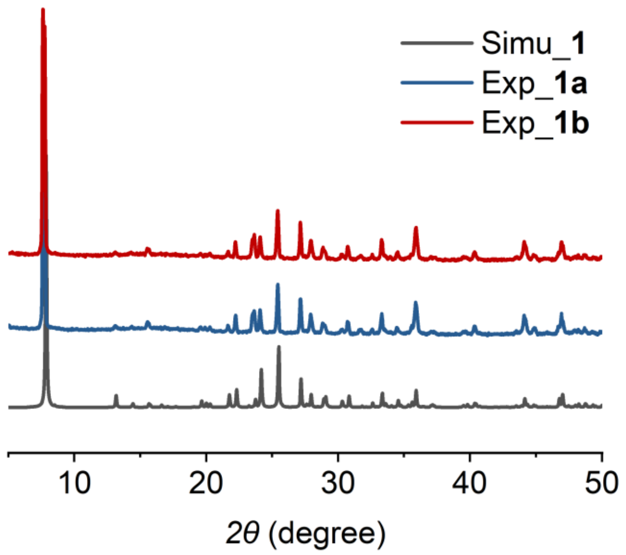

Figure S1. PXRD patterns of **1**. Experimental (Exp) and simulated (Simu) PXRD patterns of **1**.

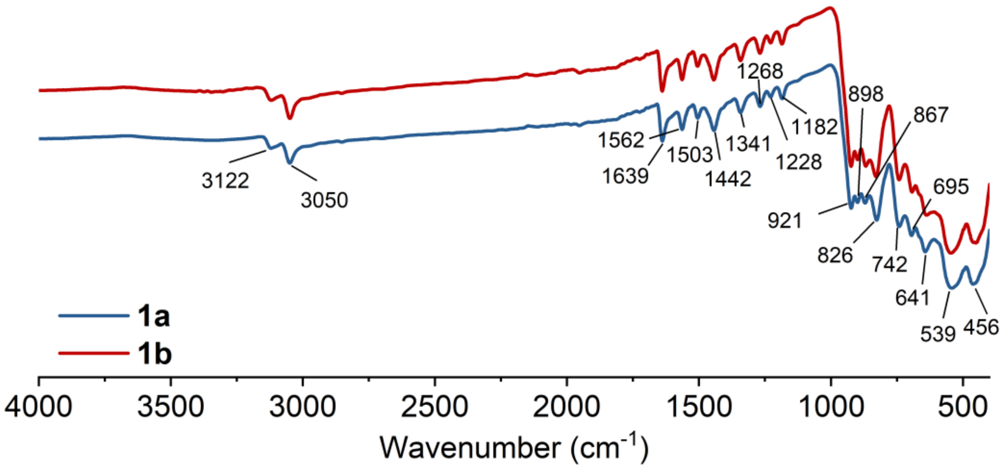

Figure S2. IR spectra of **1**. FT-IR spectra of the as-synthesized and colored samples, respectively.

Peaks at 3122, 3050 cm^{-1} ascribe to the ν(=C-H); peaks at 1639, 1562, 1503 cm^{-1} ascribe to the ν(pyridine skeleton); peaks at 1442 cm^{-1} ascribe to the δ(C-H) in $-CH_3$; peaks at positions 1350-1100 cm^{-1} mainly ascribe to the ν(C-N); the peaks below 1000 cm^{-1} mainly ascribe to the ν(Mo-O), and partly δ(= C-H).

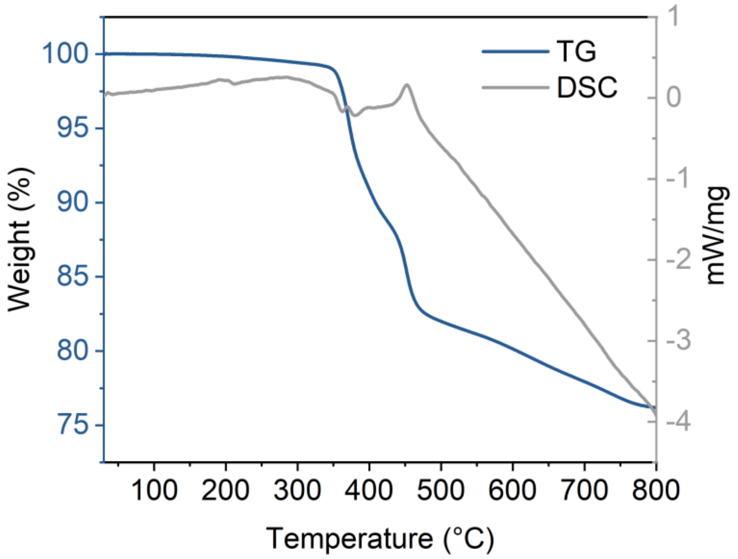

Figure S3. Thermogravimetric analysis of **1**. Thermogravimetric analysis in a nitrogen atmosphere with the ramp rate of 10 °C/min.

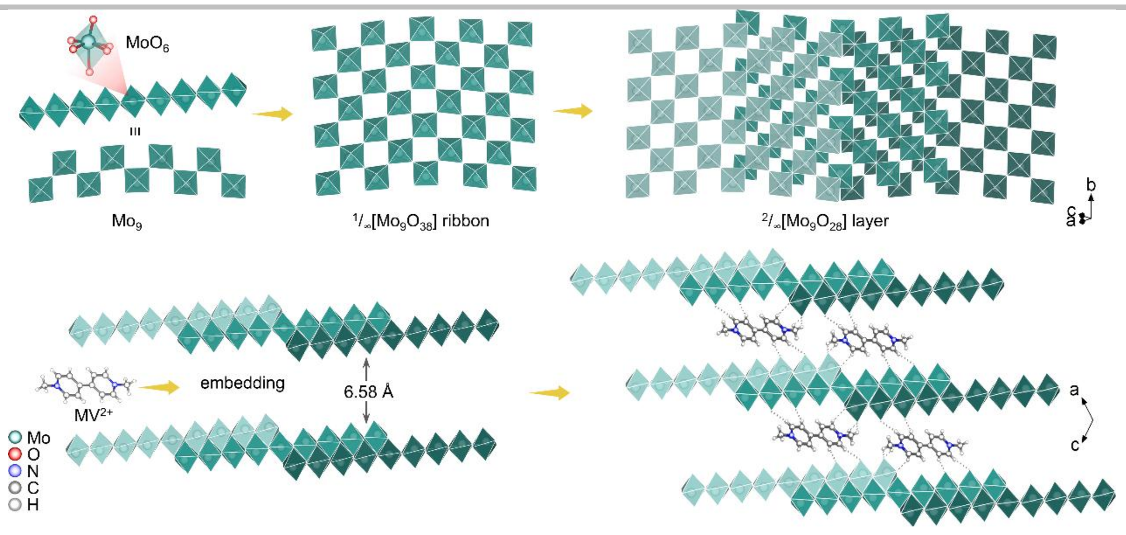

Figure S4. Structural evolution of **1**.

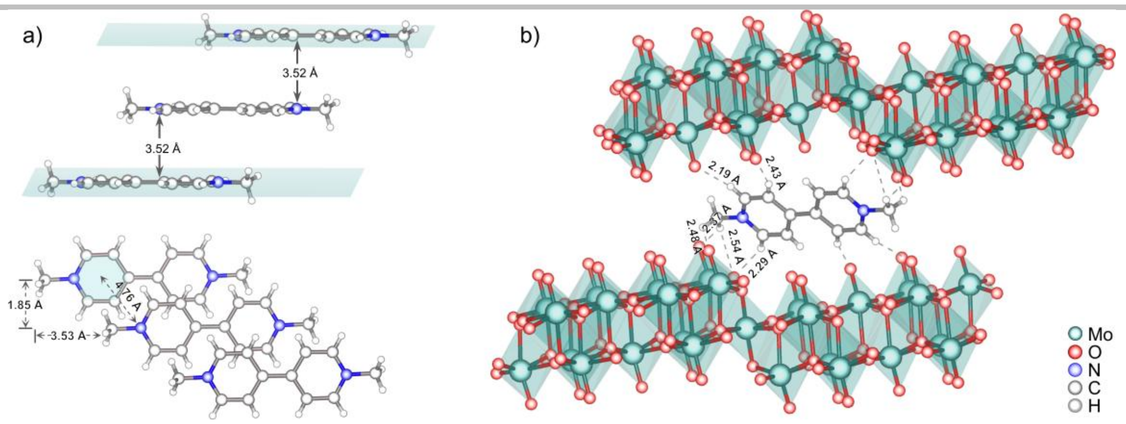

Figure S5. Intermolecular interactions of **1**. The interaction between MV^{2+} (a) and local hydrogen bonds between the molybdate layer and MV^{2+}(b).

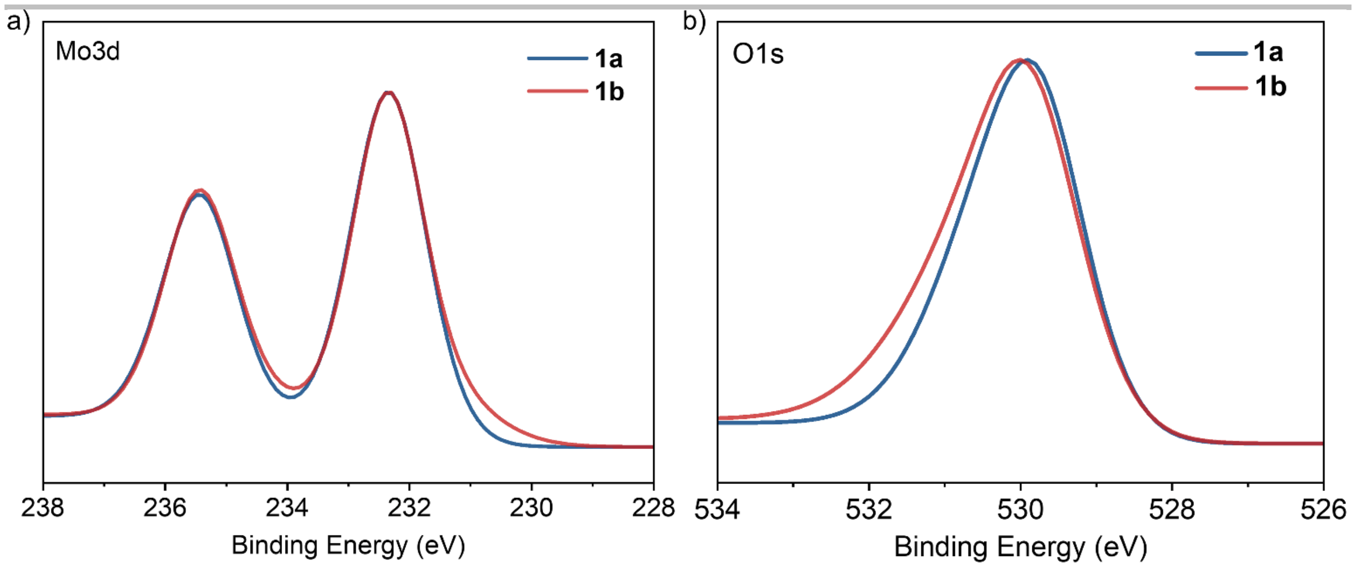

c)

**1b**	Area ratio
$Mo^{VI}\ 3d_{5/2}$	1
$Mo^{VI}\ 3d_{3/2}$	0.66
$Mo^{V}\ 3d_{5/2}$	0.07
$Mo^{V}\ 3d_{3/2}$	0.05

Figure S6. The XPS curves of Mo3d and O1s.The normalized XPS curves of Mo3d (a) and O1s (b) for **1a** and **1b**, c) The area ratio of Mo3d for Mo^{VI} and Mo^{V} in **1b**.

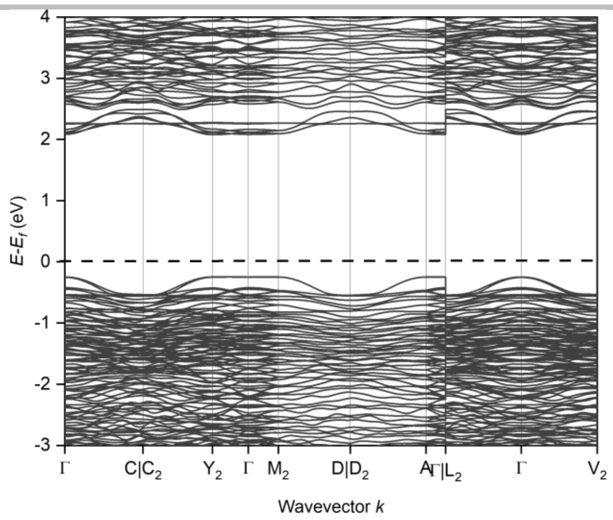

Figure S7. The band structure (BS) of **1**.The Fermi level is set to zero.

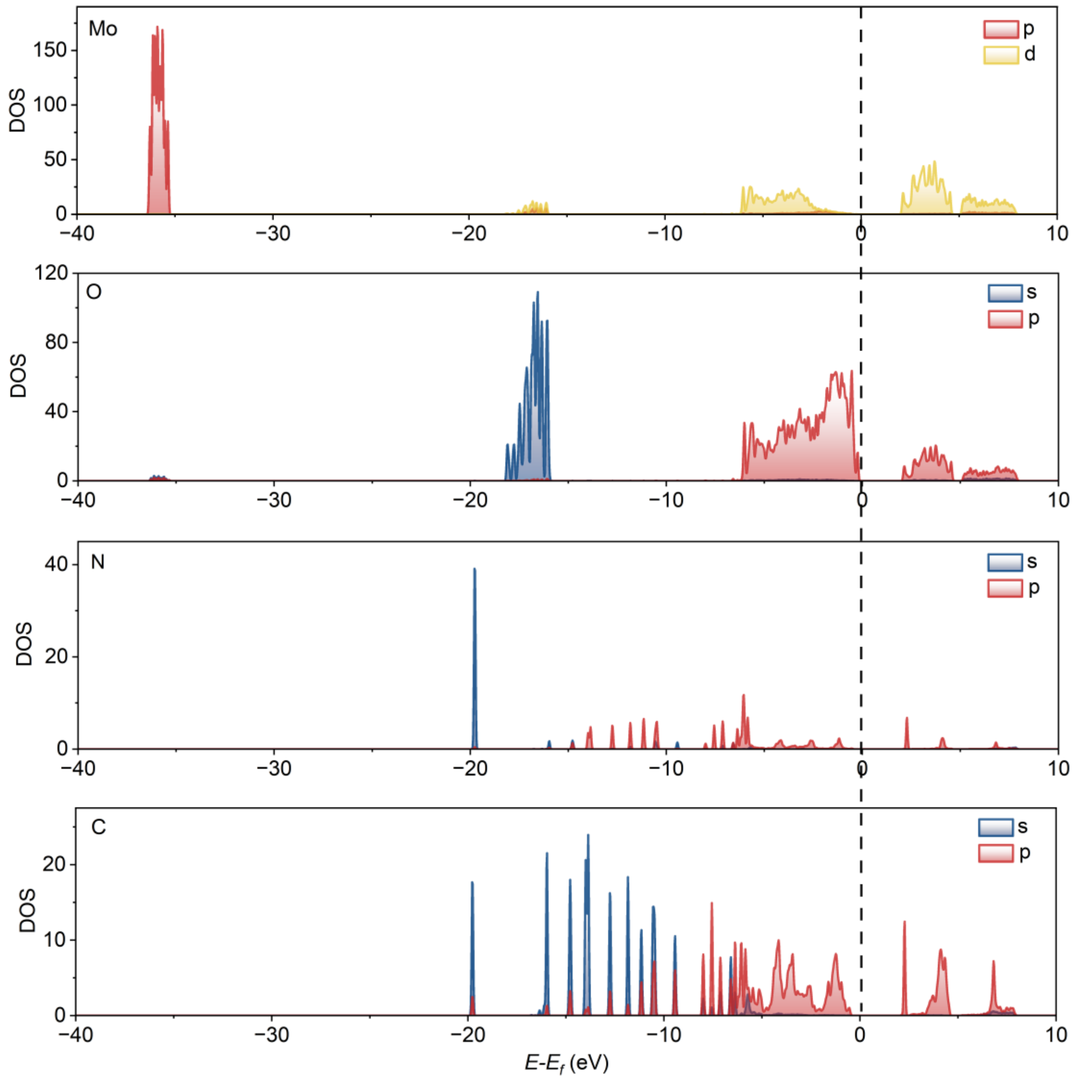

Figure S8. The partial density of states (PDOS).PDOS for Mo atoms, O atoms, N atoms and C atoms.The Fermi level is set to zero.

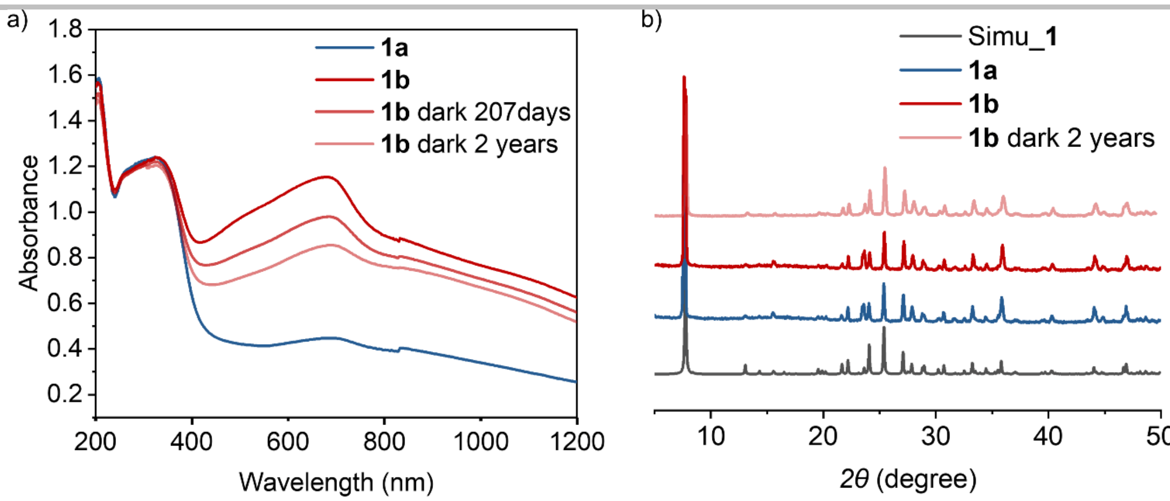

Figure S9. Stability test of charge-separated state of **1**.The UV-Vis spectra (a) and PXRD (b) for **1a**, **1b** and **1b** dark 2 years.

The absorption bands after coloration for up to two years storage under ambient laboratory conditions (Fuzhou, China; estimated range: ~5-35 °C, 60-90% RH; in the dark) still have strong absorption, which shows that the sample is still in the colored state, indicating that the charge-separated state has extremely high stability.

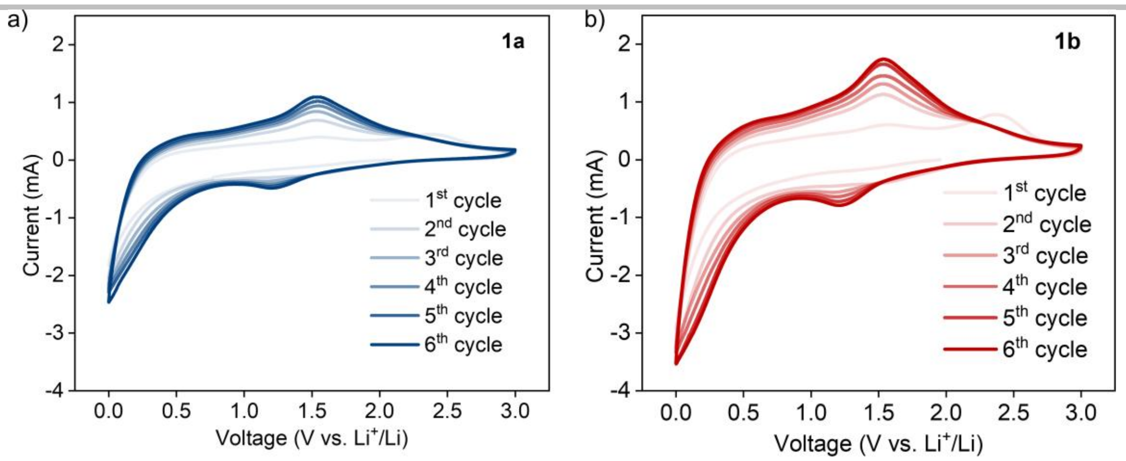

Figure S10. CV curves at 1.0 mV s^{-1}.**1a** (a) and **1b** (b) in the initial six cycles at 1.0 mV s^{-1}.

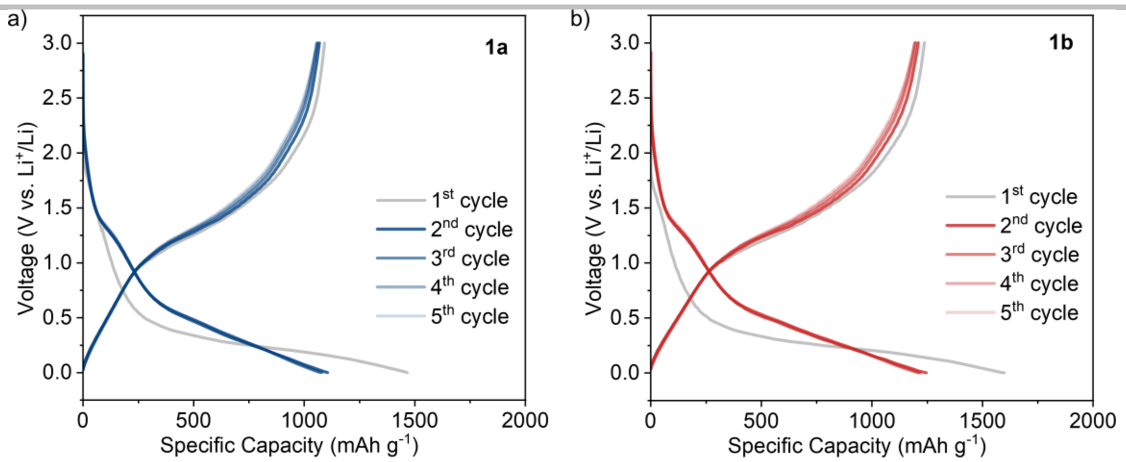

Figure S11. The first five cycles galvanostatic charge-discharge curves.(a) **1a** and (b) **1b** at a current density of 0.1 A g^{-1}.

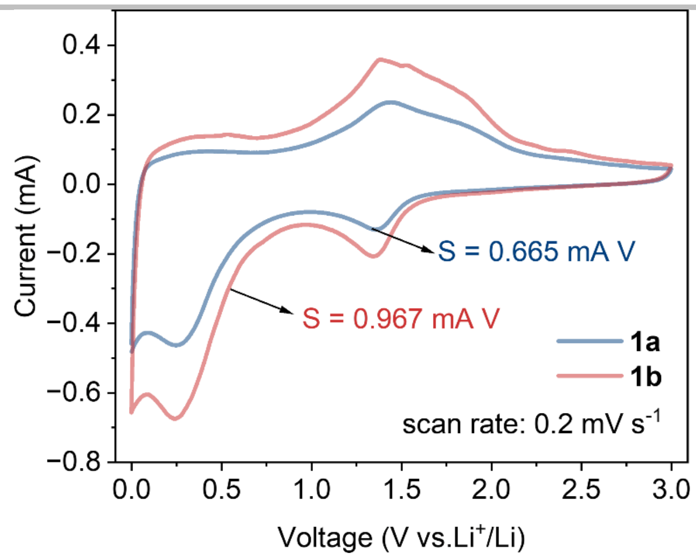

Figure S12. CV comparison of **1a** and **1b** at a scan rate of 0.2 mV s^{-1}.

The specific capacity of **1a** is 1034 mAhg^{-1} and that of **1b** is 1230 mAhg^{-1}. Calculations based on the relative molecular masses of **1** show that **1a** can embed 57 Li^+ and **1b** can embed 68 Li^+.

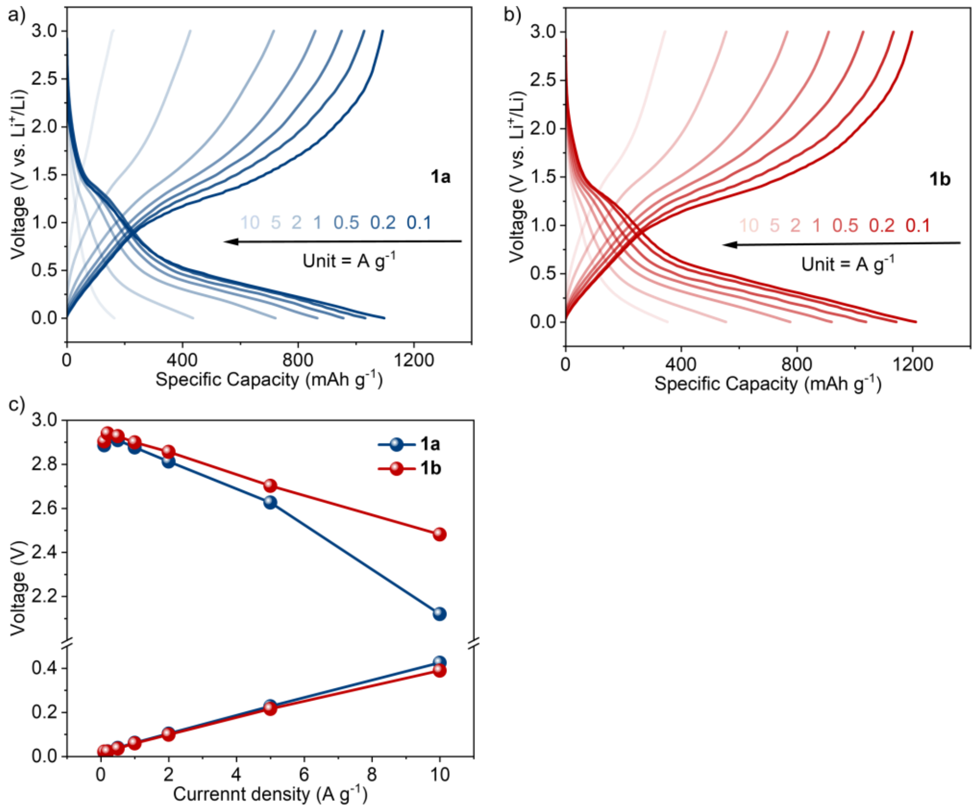

Figure S13. Charge-discharge curves.Charging/discharging profiles of **1a** (a) and **1b** (b) electrode at current densities of 0.1, 0.2, 0.5, 1, 2, 5 and 10 Ag^{-1}. (c) The initial voltages of lithiation/delithiation at different current density for **1a** and **1b** electrodes.

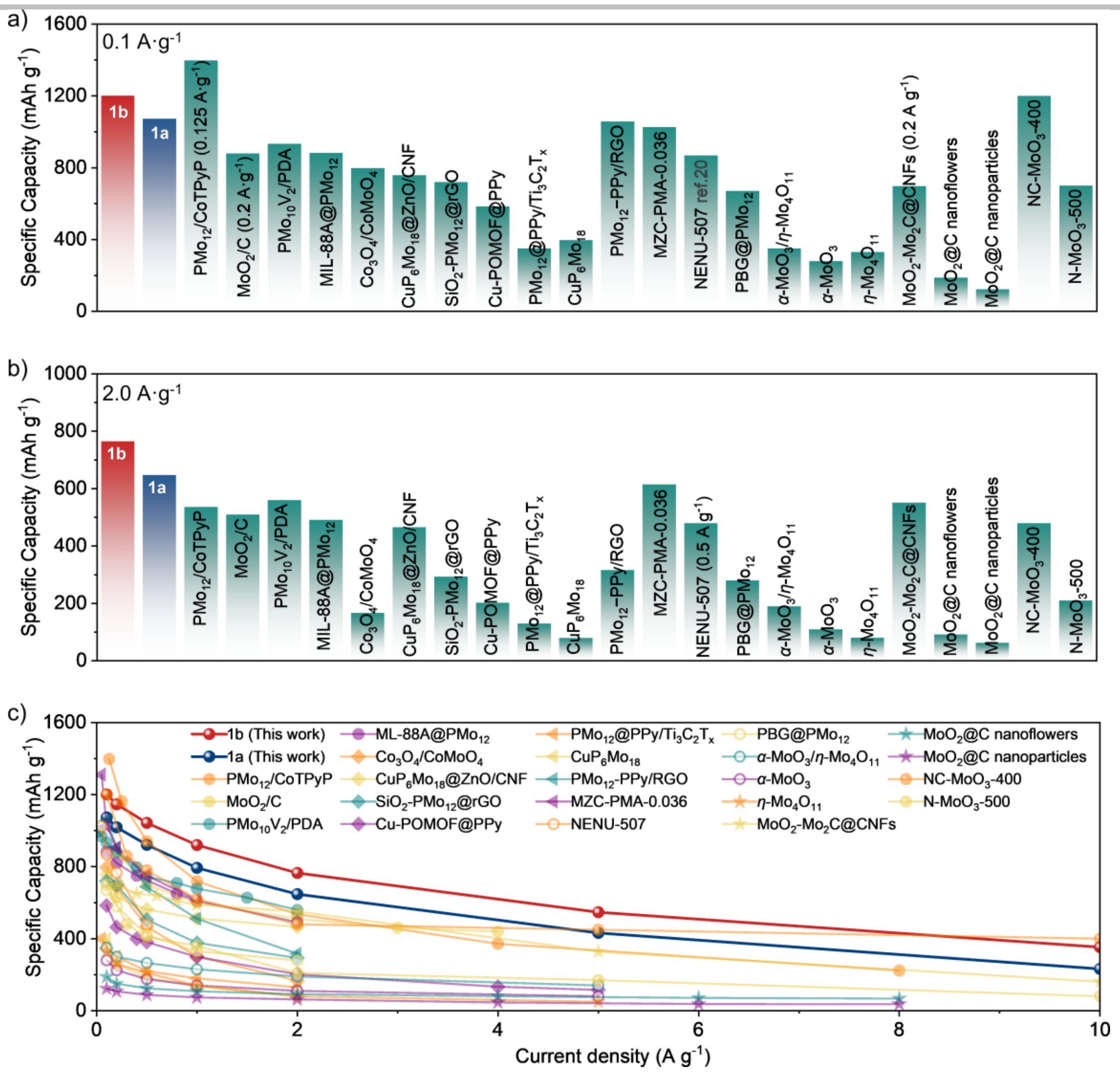

Figure S14. Comparison of specific capacities with reported literature.0.1 A g-1 (a), 2 A g-1 (b) and different current densities (c) for molybdates.

Note: $PMo_{12}/CoTPyP$ ref.9; MoO_2/C ref.10; $PMo_{10}V_2/PDA$ ref.11; MIL-88A@PMo_{12} ref.12; $Co_3O_4/CoMoO_4$ ref.13; CuP_6Mo_{18}@ZnO/CNFs ref.14; SiO_2-PMo_{12}@rGO ref.15; Cu-POMOF@PPy ref.16; PMo_{12}@PPy/$Ti_3C_2T_x$ ref.17; CuP_6Mo_{18} ref.14; PMo_{12}–PPy/RGO ref.18; MZC-PMA-0.036 ref.19; NENU-507 ref.20; PBG@PMo_{12} ref.21; α-MoO_3/η-Mo_4O_{11}; α-MoO_3 and η-Mo_4O_{11} ref.22; MoO_2-Mo_2C@CNFs ref.23; MoO_2@C nanoflowers and MoO_2@C nanoparticles ref.24; NC-MoO_3-400 and N-MoO_3-500 ref.25.

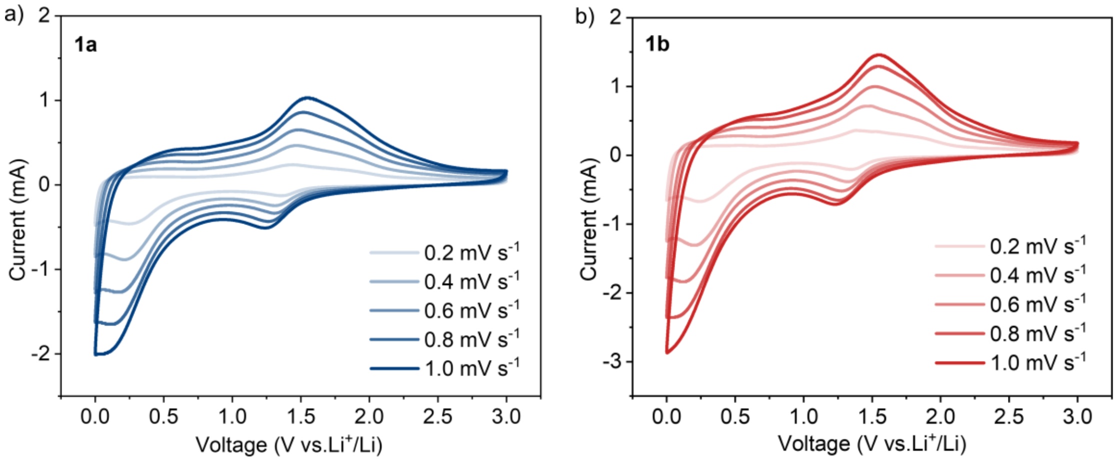

Figure S15. CV curves at different scan rates.**1a** (a) and **1b (b)** at scan rates from 0.2 to 1.0 mV s^{-1}.

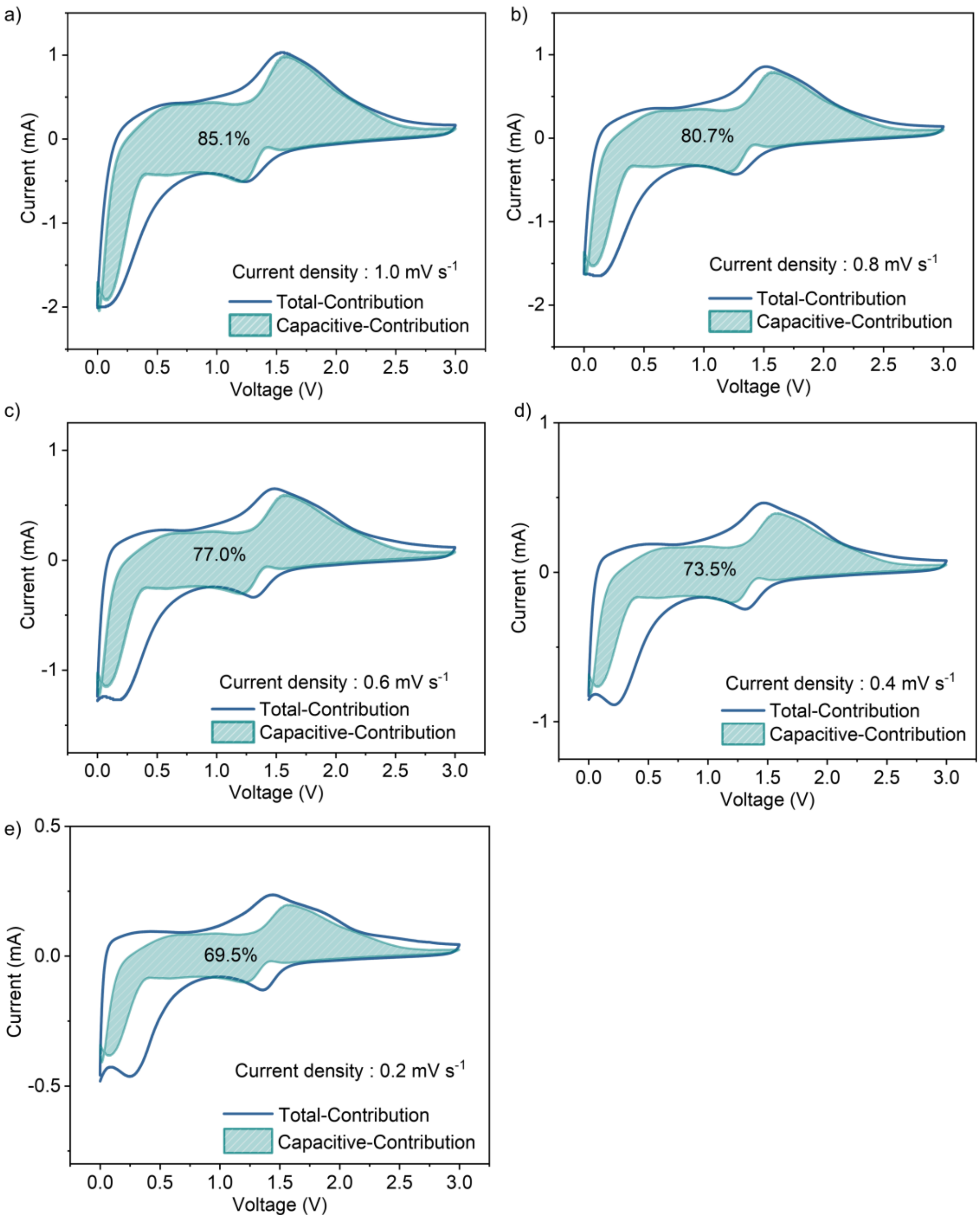

Figure S16. Calculated total current and capacitive current of **1a.1a** from the CVs at scan rates of 1.0 (a), 0.8 (b), 0.6 (c), 0.4 (d) and 0.2 mV s^{-1} (e), respectively.

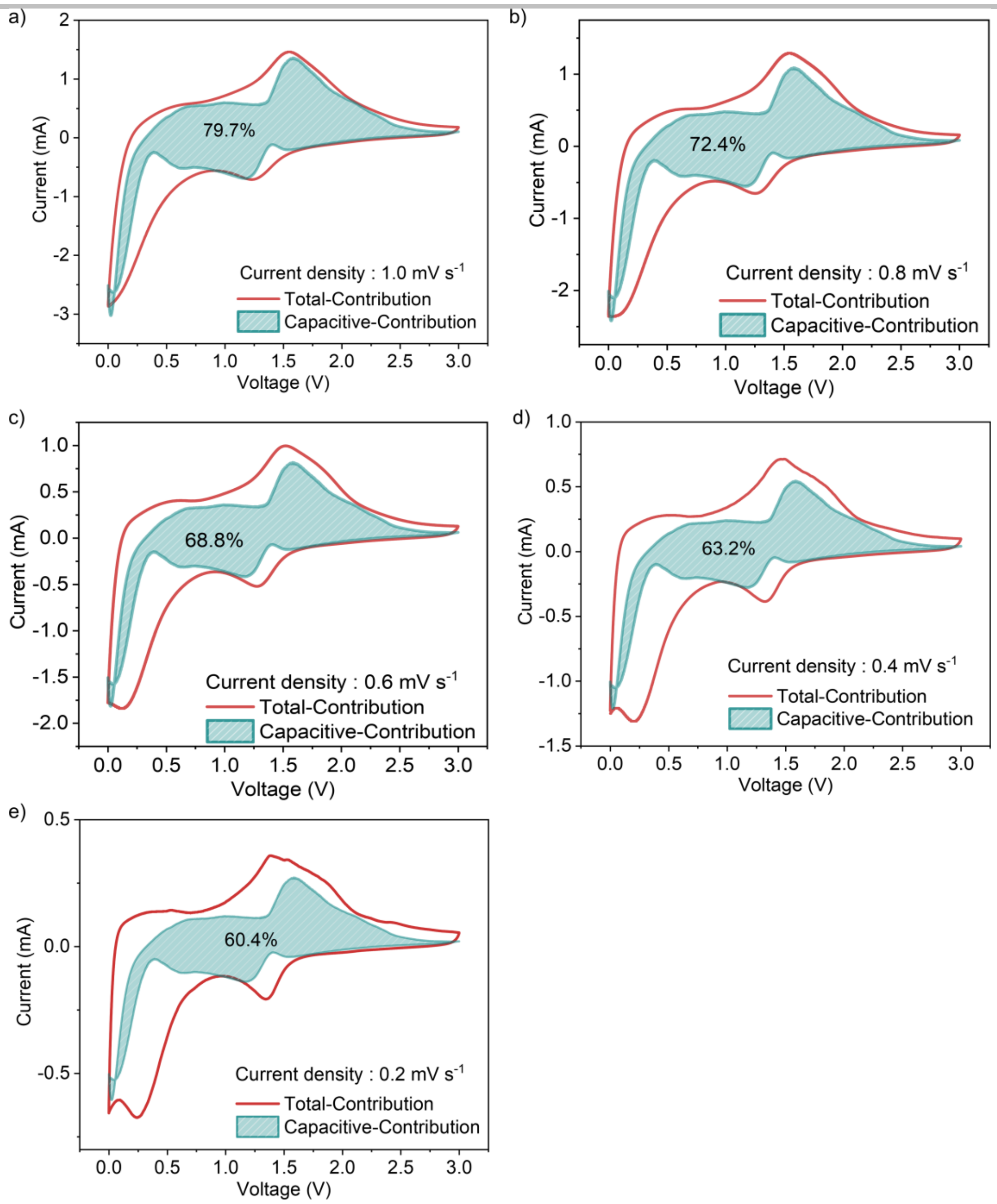

Figure S17. Calculated total current and capacitive current of **1b.1b** from the CVs at scan rates of 1.0 (a), 0.8 (b), 0.6 (c), 0.4 (d) and 0.2 mV s^{-1} (e), respectively.

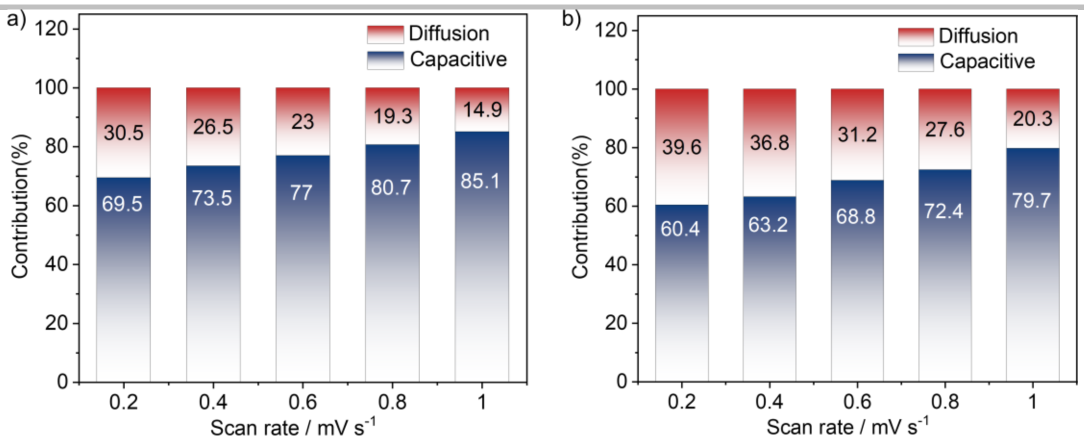

Figure S18. Column diagram of the capacitive ratio at various scan rates.(0.2, 0.4, 0.6, 0.8 and 1.0 mV s^{-1}) for **1a** (a) and **1b** (b).

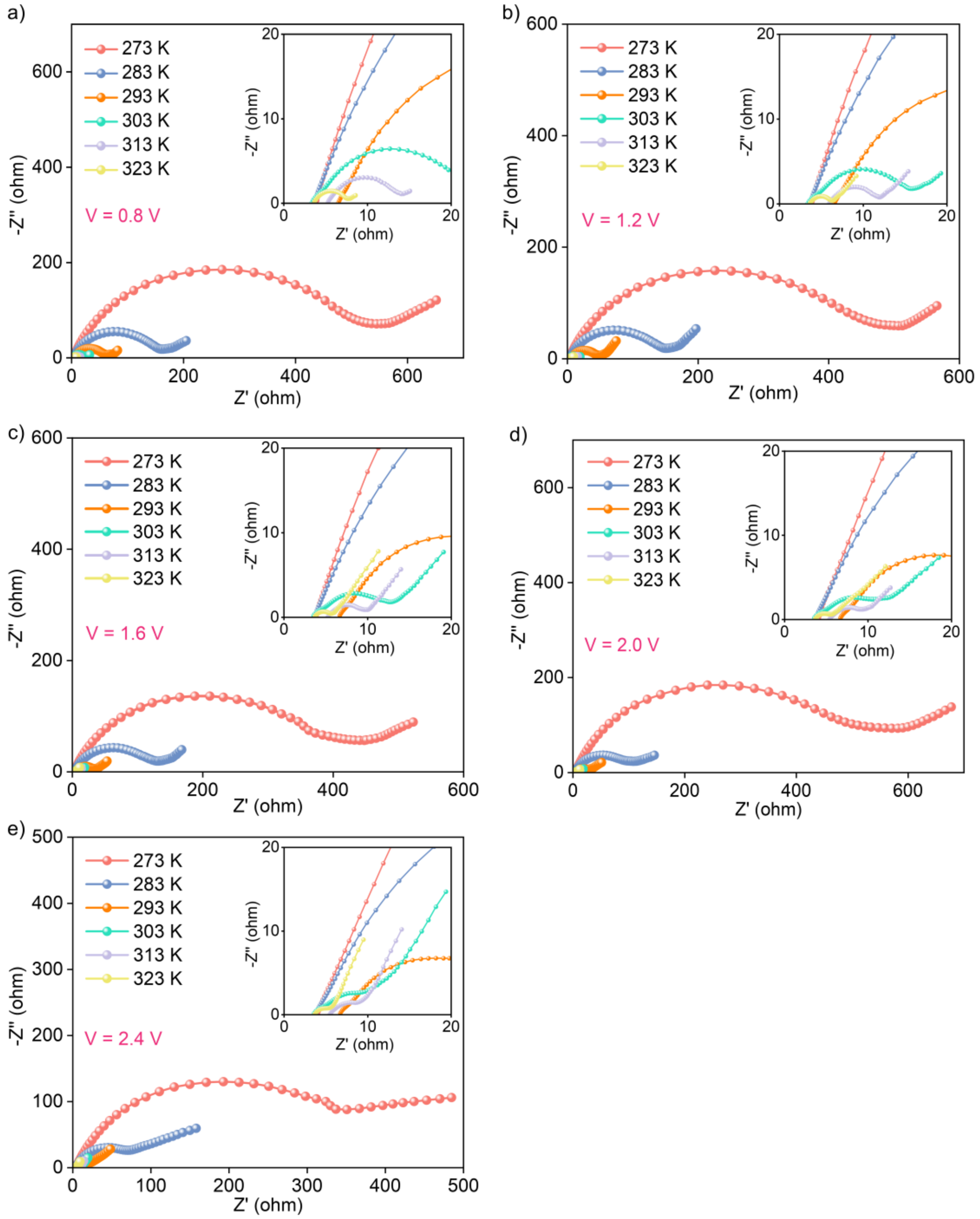

Figure S19. Nyquist plots of **1a**.Nyquist plots of **1a** at 0.8 (a), 1.2 (b), 1.6 (c), 2.0 (d) and 2.4V (e), respectively (the inset is the zoomed-out graph).

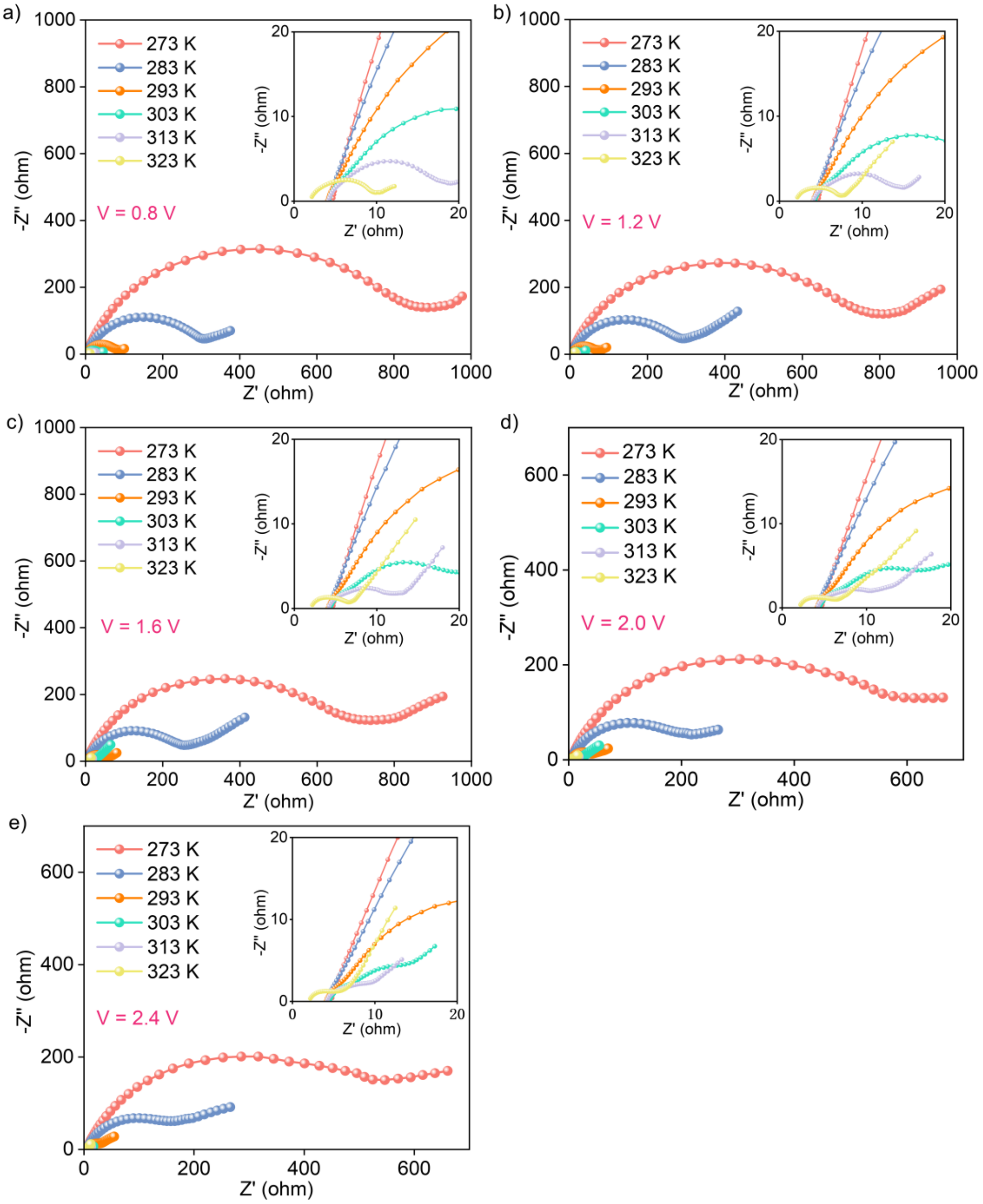

Figure S20. Nyquist plots of **1b**.Nyquist plots of **1b** at 0.8 (a), 1.2 (b), 1.6 (c), 2.0 (d) and 2.4V (e), respectively (the inset is the zoomed-out graph).

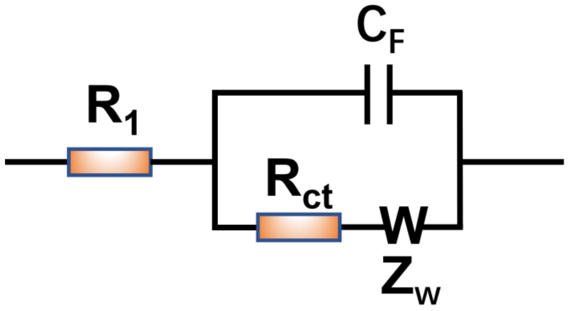

Figure S21. The equivalent circuit. The equivalent circuit is used in fitting the electrochemical impedance spectroscopy (EIS).

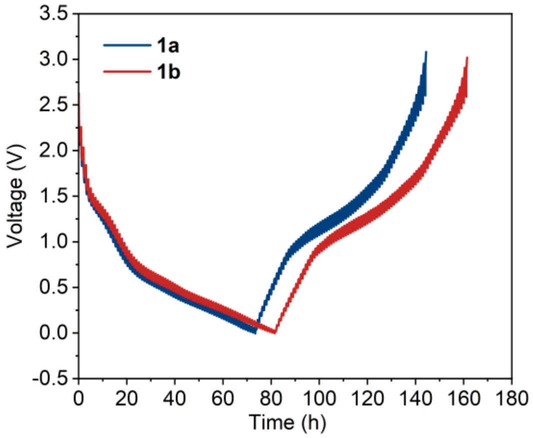

Figure S22. GITT curves.**1a** and **1b** electrode at 0.05 A g^{-1} after the sixth cycle.

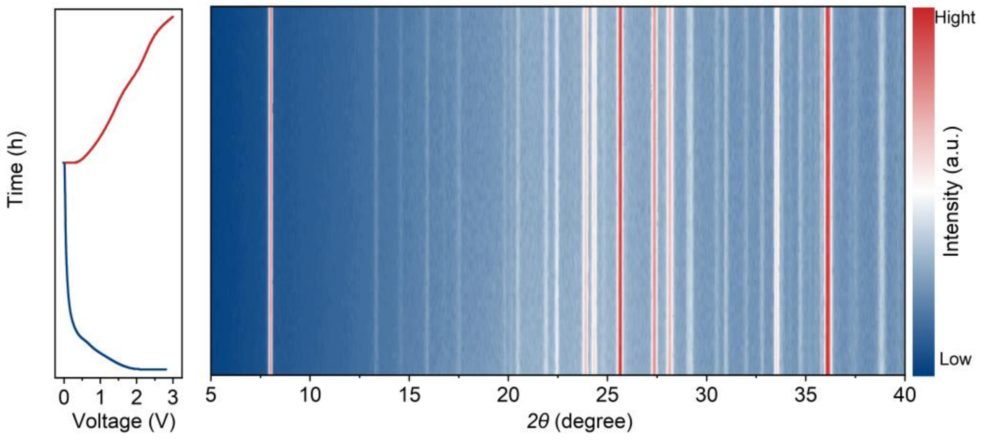

Figure S23. *In-situ* XRD patterns of **1b**.**1b** electrode at different charge/discharge stages in the first cycle.

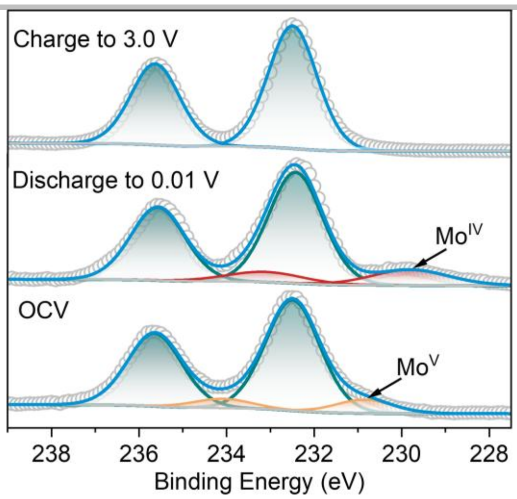

Figure S24. Mo 3d XPS spectra of **1b**..Mo 3d XPS spectra of **1b** at the pristine and fully discharged (0.01 V) and charged (3 V) states.

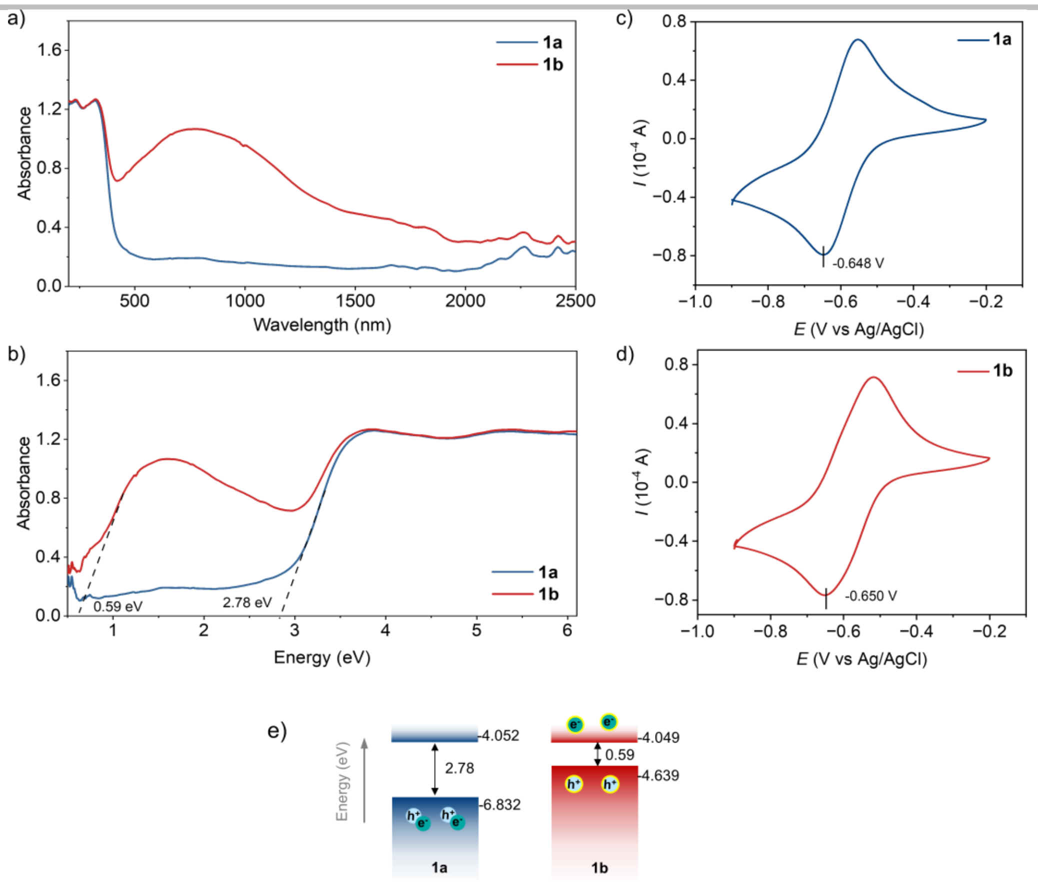

Figure S25. Energy levels of **1**.(a, b) UV-Vis-NIR absorption spectrum; (c, d) CV curves at a scan rate of 100 mV/s in 0.1 M KOH; (e) Schematic diagram of energy band structure of 1a and 1b.

The working electrode was a homemade glassy carbon electrode with a diameter of 3 mm. Before each use, it is carefully polished with aluminum oxide powder (50 nm), rinsed with ethanol, and dried. The counter electrode was a Platinum wire electrode. The reference electrode is Ag/AgCl electrode.

The absorption spectrum of **1a** and **1b** was used to calculate the band gap energy (E_g) (Figure S24a) **1a** exhibits an E_g of 2.78 eV, whereas **1b** possesses a significantly narrower E_g of 0.59 eV. (Figure S24b). CV measurements established that the reduction potentials of **1a** and **1b** are −0.648 V and −0.650 V (vs Ag/AgCl), respectively (Figure S24c, d). Given that the potential of the Ag/AgCl reference electrode corresponds to -4.7 eV relative to the vacuum level,[26] the valence band and conduction band edge positions of **1a** and **1b** were determined via the following equations:

$$E_{CB} = E_{ref} - E_{red} \text{ (eV)}$$

$$E_{VB} = E_{CB} - E_g \text{(eV)}$$

Where E_{CB} and E_{VB} denote the conduction band (CB) edge and valence band (VB) edge, respectively, E_{ref} represents the potential of the reference electrode relative to the vacuum level, E_{red} is the reduction potential. (Figure S24e).

Then, CV curves combined with band energies were used to confirm the energy bond structure of **1a** and **1b**. The CB and VB positions of **1a** are positioned at -4.052 eV and -6.832 eV, respectively, while those of **1b** reside at -4.049 eV and -4.639 eV.

References

[1] X. Hu.; M. Qiu.; Y. Liu.; J. Yuan.; J. Chen.; H. Zhan; Z. Wen. Interface and Structure Engineering of Tin-Based Chalcogenide Anodes for Durable and Fast-Charging Sodium Ion Batteries. *Adv. Energy Mater.* **2022**, *12*, 2202318-2202325.

[2] X. C. Ren.; J. S. Wang.; D. M. Zhu.; Q. W. Li.; W. F. Tian.; L. Wang.; J. B. Zhang.; L. Miao.; P. K. Chu; K. F. Huo. Sn-C bonding riveted SnSe nanoplates vertically grown on nitrogen-doped carbon nanobelts for high-performance sodium-ion battery anodes. *Nano Energy* **2018**, *54*, 322-330.

[3] W. Wang.; P. Li.; H. Zheng.; Q. Liu.; F. Lv.; J. Wu.; H. Wang; S. Guo. Ultrathin Layered SnSe Nanoplates for Low Voltage, High-Rate, and Long-Life Alkali-Ion Batteries. *Small* **2017**, *13*, 1702228-1702234.

[4] J. Hafner. Ab-initiosimulations of materials using VASP: Density-functional theory and beyond. *J. Comput. Chem.* **2008**, *29*, 2044-2078.

[5] C.-Y. Pan.; X.-R. Yang.; L. Xiong.; Z.-W. Lu.; B.-Y. Zhen.; X. Sui.; X.-B. Deng.; L. Chen; L.-M. Wu. Solid-State Nonlinear Optical Switch with the Widest Switching Temperature Range Owing to Its Continuously Tunable Tc. *J. Am. Chem. Soc.* **2020**, *142*, 6423-6431.

[6] T. D. Kuehne.; M. Iannuzzi.; M. Del Ben.; V. V. Rybkin.; P. Seewald.; F. Stein.; T. Laino.; R. Z. Khaliullin.; O. Schutt.; F. Schiffmann.; D. Golze.; J. Wilhelm.; S. Chulkov.; M. H. Bani-Hashemian.; V. Weber.; U. Borstnik.; M. Taillefumier.; A. S. Jakobovits.; A. Lazzaro.; H. Pabst.; T. Mueller.; R. Schade.; M. Guidon.; S. Andermatt.; N. Holmberg.; G. K. Schenter.; A. Hehn.; A. Bussy.; F. Belleflamme.; G. Tabacchi.; A. Gloss.; M. Lass.; I. Bethune.; C. J. Mundy.; C. Plessl.; M. Watkins.; J. VandeVondele.; M. Krack; J. Hutter. CP2K: An electronic structure and molecular dynamics software package - Quickstep: Efficient and accurate electronic structure calculations. *J. Chem. Phys.* **2020**, *152*, 194103.

[7] K. Momma; F. Izumi. VESTA: a three-dimensional visualization system for electronic and structural analysis. *J. Appl. Crystallogr.* **2008**, *41*, 653-658.

[8] G. Henkelman.; B. P. Uberuaga; H. Jónsson. A climbing image nudged elastic band method for finding saddle points and minimum energy paths. *J. Chem. Phys.* **2000**, *113*, 9901-9904.

[9] Y. Liu.; X. Zhou.; T. Qiu.; R. Yao.; F. Yu.; T. Song.; X. Lang.; Q. Jiang.; H. Tan.; Y. Li; Y. Li. Co-Assembly of Polyoxometalates and Porphyrins as Anode for High-Performance Lithium-Ion Batteries. *Adv. Mater.* **2024**, *36*, 2407705-2407715.

[10] Y. Wang.; L. Yu; X. W. Lou. Formation of Triple-Shelled Molybdenum–Polydopamine Hollow Spheres and Their Conversion into MoO_2/Carbon Composite Hollow Spheres for Lithium-Ion Batteries. *Angew. Chem. Int. Ed.* **2016**, *55*, 14668-14672.

[11] Y. H. Ding.; J. Peng.; S. U. Khan; Y. Yuan. A New Polyoxometalate (POM)-Based Composite: Fabrication through POM-Assisted Polymerization of Dopamine and Properties as Anode Materials for High-Performance Lithium-Ion Batteries. *Chem. - Eur. J.* **2017**, *23*, 10338-10343.

[12] X. Zhao.; G. Niu.; H. Yang.; J. Ma.; M. Sun.; M. Xu.; W. Xiong.; T. Yang.; L. Chen; C. Wang. MIL-88A@polyoxometalate microrods as an advanced anode for high-performance lithium ion batteries. *CrystEngComm* **2020**, *22*, 3588-3597.

[13] X.-L. Yang.; Y.-S. Ye.; Z.-M. Wang.; Z.-H. Zhang.; Y.-L. Zhao.; F. Yang.; Z.-Y. Zhu; T. Wei. POM-Based MOF-Derived Co_3O_4/$CoMoO_4$ Nanohybrids as Anodes for High-Performance Lithium-Ion Batteries. *ACS Omega* **2020**, *5*, 26230-26236.

[14] S. Ge.; L. Cui.; K. Yu.; M. Wang.; L. Guo; B. Zhou. Basket-like POMs and ZnO co-modified CNFs as anode of high-efficient Lithium-ion batteries. *J. Energy Storage* **2024**, *93*, 112312-112321.

[15] H. Hu.; X. Jia.; J. Wang.; W. Chen.; L. He; Y.-F. Song. Confinement of PMo12 in hollow SiO_2-PMo_{12}@rGO nanospheres for high-performance lithium storage. *Inorg. Chem. Front.* **2021**, *8*, 352-360.

[16] Z. Han.; X. Li.; Q. Li.; H. Li.; J. Xu.; N. Li.; G. Zhao.; X. Wang.; H. Li; S. Li. Construction of the POMOF@Polypyrrole Composite with Enhanced Ion Diffusion and Capacitive Contribution for High-Performance Lithium-Ion Batteries. *ACS Appl. Mater. Interfaces* **2021**, *13*, 6265-6275.

[17] M. Mahajan.; G. Singla; S. Ogale. Polypyrrole-Encapsulated Polyoxomolybdate Decorated MXene As a Functional 2D/3D Nanohybrid for a Robust and High Performance Li-Ion Battery. *ACS Appl. Energy Mater.* **2021**, *4*, 4541-4550.

[18] M. Zhang.; T. Wei.; A. M. Zhang.; S.-L. Li.; F.-C. Shen.; L.-Z. Dong.; D.-S. Li; Y.-Q. Lan. Polyoxomolybdate–Polypyrrole/Reduced Graphene Oxide Nanocomposite as High-Capacity Electrodes for Lithium Storage. *ACS Omega* **2017**, *2*, 5684-5690.

[19] Z. M. Yang.; S. P. Zhao.; M. H. Zhang.; Z. D. Zhang.; T. R. Ma.; S. Yuan.; J. Su.; C. H. Li; J. L. Zuo. Coordination-Modulated Metal Tetrathiafulvalene Octacarboxylate Frameworks for High-Performance Lithium-Ion Battery Anodes. *Angew. Chem. Int. Ed.* **2023**, *62*, e202304183.

[20] Y.-Y. Wang.; M. Zhang.; S.-L. Li.; S.-R. Zhang.; W. Xie.; J.-S. Qin.; Z.-M. Su; Y.-Q. Lan. Diamondoid-structured polymolybdate-based metal–organic frameworks as high-capacity anodes for lithium-ion batteries. *Chem. Commun.* **2017**, *53*, 5204-5207.

[21] H. Cui.; S. Li.; J. Zhuo.; J. Sha; M. Hu. Keggin-type phosphomolybdate anchored paper ball-like graphene as high-capacity anode material for lithium-ion batteries. *Solid State Sci.* **2023**, *138*, 107132-107139.

[22] S. Sun.; Z. Han.; W. Liu.; Q. Xia.; L. Xue.; X. Lei.; T. Zhai.; D. Su; H. Xia. Lattice pinning in MoO3 via coherent interface with stabilized Li^+ intercalation. *Nat. Commun.* **2023**, *14*, 6662-6675.

[23] S. Zhu.; J. Fan.; Y. Yang.; L. You; X. Wu. MoO_2-Mo_2C uniformly encapsulated into N, P co-doped carbon nanofibers as a freestanding anode for high and long-term lithium storage. *J. Electroanal. Chem.* **2022**, *917*, 116414-116421.

[24] C. Cui.; Q. Wei.; L. Zhou.; L. Mai; J. Ma. Facile synthesis of MoO_2@C nanoflowers as anode materials for sodium-ion batteries. *Mater. Res. Bull.* **2017**, *94*, 122-126.
[25] M. Guo.; L. Huang.; C. Zhao.; L. He.; Y. Wang.; G. Dou.; G. Zhang; X. Sun. Atomic Mo-NC-sourced robust MoO_3/C nanocomposite for high-performance Li-ion storage. *Sci. China Mater.* **2023**, *66*, 3054-3064.
[26] M. R. Andersson.; M. Berggren.; O. Inganaes.; G. Gustafsson.; J. C. Gustafsson-Carlberg.; D. Selse.; T. Hjertberg; O. Wennerstroem. Electroluminescence from Substituted Poly (thiophenes): From Blue to Near-Infrared. *Macromolecules* **1995**, *28*, 7525-7529.
