# Supplementary material for: Photochromic Molybdate for Advancing Anode Capacity of Lithium‐Ion Battery
Source: Adv Sci (Weinh). 2025 Nov 30;13(6):e19866. doi: 10.1002/advs.202519866 (PMC12866782; doi:10.1002/advs.202519866)

checkCIF/PLATON report

Structure factors have been supplied for datablock(s) a

THIS REPORT IS FOR GUIDANCE ONLY. IF USED AS PART OF A REVIEW PROCEDURE FOR PUBLICATION, IT SHOULD NOT REPLACE THE EXPERTISE OF AN EXPERIENCED CRYSTALLOGRAPHIC REFEREE.

No syntax errors found. CIF dictionary Interpreting this report

Datablock: a

Bond precision: C-C = 0.0110 A Wavelength=0.71073

Cell:	a=26.8624(16)	b=5.4839(2)	c=24.6339(11)
	alpha=90	beta=122.636(6)	gamma=90
Temperature:	293 K		

	Calculated	Reported
Volume	3055.9(3)	3055.9(3)
Space group	C 2/c	C 1 2/c 1
Hall group	-C 2yc	-C 2yc
Moiety formula	Mo9 O28, 2(C6 H7 N)	Mo9 O28, C12 H14 N2
Sum formula	C12 H14 Mo9 N2 O28	C12 H14 Mo9 N2 O28
Mr	1497.71	1497.71
Dx,g cm-3	3.255	3.255
Z	4	4
Mu (mm-1)	3.678	3.678
F000	2808.0	2808.0
F000'	2747.19	
h,k,lmax	31,6,29	31,6,29
Nref	2709	2678
Tmin,Tmax		
Tmin'		

Correction method= Not given

Data completeness= 0.989 Theta(max)= 25.022

R(reflections)= 0.0316(2245) wR2(reflections)= 0.0895(2678)

S = 1.082 Npar= 240

The following ALERTS were generated. Each ALERT has the format
test-name_ALERT_alert-type_alert-level.
Click on the hyperlinks for more details of the test.

Alert level B

```
PLAT971_ALERT_2_B Check Calcd Resid. Dens.  2.01Ang From C1              2.74 eA-3
PLAT973_ALERT_2_B Check Calcd Positive Resid. Density on       Mo2       1.85 eA-3
PLAT973_ALERT_2_B Check Calcd Positive Resid. Density on       Mo3       1.60 eA-3
```

Alert level C

```
PLAT042_ALERT_1_C Calc. and Reported MoietyFormula Strings  Differ     Please Check
              Calc: Mo9 O28, 2(C6 H7 N)
              Rep.: Mo9 O28, C12 H14 N2
PLAT053_ALERT_1_C Minimum Crystal Dimension Missing (or Error) ...     Please Check
PLAT054_ALERT_1_C Medium  Crystal Dimension Missing (or Error) ...     Please Check
PLAT055_ALERT_1_C Maximum Crystal Dimension Missing (or Error) ...     Please Check
PLAT094_ALERT_2_C Ratio of Maximum / Minimum Residual Density ....       2.93 Report
PLAT223_ALERT_4_C Solv./Anion  Resd 2  H   Ueq(max)/Ueq(min) Range        5.9 Ratio
PLAT245_ALERT_2_C U(iso) H4        Smaller than U(eq) C4        by      0.024 Ang**2
PLAT342_ALERT_3_C Low Bond Precision on  C-C Bonds ..............       0.011 Ang.
PLAT350_ALERT_3_C Short  C-H (X0.96,N1.08A)  C4       - H4       .       0.84 Ang.
PLAT911_ALERT_3_C Missing FCF Refl Between Thmin & STh/L=    0.595         28 Report
               16  0  6,  14  0  8,  16  0  8, -28  0 10,  12  0 10,  14  0 10,
               16  0 10,  10  0 12,  12  0 12,  14  0 12,  16  0 12,   8  0 14,
               10  0 14,  12  0 14,  14  0 14,   6  0 16,   8  0 16,  10  0 16,
               12  0 16,   4  0 18,   6  0 18,   8  0 18,  10  0 18,   4  0 20,
                6  0 20,   2  0 22,   4  0 22,   0  0 24,
PLAT973_ALERT_2_C Check Calcd Positive Resid. Density on       Mo5       1.28 eA-3
PLAT973_ALERT_2_C Check Calcd Positive Resid. Density on       Mo4       1.05 eA-3
PLAT975_ALERT_2_C Check Calcd Resid. Dens.  0.42Ang From O14     .       0.65 eA-3
PLAT975_ALERT_2_C Check Calcd Resid. Dens.  0.97Ang From O14     .       0.55 eA-3
PLAT975_ALERT_2_C Check Calcd Resid. Dens.  0.40Ang From O3      .       0.47 eA-3
```

Alert level G

```
PLAT004_ALERT_5_G Polymeric Structure Found with Maximum Dimension          2 Info
PLAT083_ALERT_2_G SHELXL Second Parameter in WGHT  Unusually Large      23.01 Why ?
PLAT128_ALERT_4_G Alternate Setting for Input Space Group     C2/c       I2/a Note
PLAT164_ALERT_4_G Nr. of Refined C-H H-Atoms in Heavy-Atom Struct.          2 Note
PLAT199_ALERT_1_G Reported _cell_measurement_temperature ..... (K)        293 Check
PLAT200_ALERT_1_G Reported   _diffrn_ambient_temperature ..... (K)        293 Check
PLAT432_ALERT_2_G Short Inter X...Y Contact  O5       ..C5       .       3.01 Ang.
                                             x,-1+y,z  =      1_545 Check
PLAT432_ALERT_2_G Short Inter X...Y Contact  O13      ..C1       .       2.99 Ang.
                                       3/2-x,1/2+y,1/2-z  =      4_655 Check
PLAT794_ALERT_5_G Tentative Bond Valency for Mo1       (VI)      .       6.01 Info
PLAT794_ALERT_5_G Tentative Bond Valency for Mo2       (VI)      .       6.02 Info
PLAT794_ALERT_5_G Tentative Bond Valency for Mo3       (VI)      .       6.11 Info
PLAT794_ALERT_5_G Tentative Bond Valency for Mo4       (VI)      .       6.06 Info
PLAT794_ALERT_5_G Tentative Bond Valency for Mo5       (VI)      .       6.00 Info
PLAT909_ALERT_3_G Percentage of I>2sig(I) Data at Theta(Max) Still        77% Note
PLAT910_ALERT_3_G Missing # of FCF Reflection(s) Below Theta(Min).          3 Note
                2  0  0,  -2  0  2,   0  0  2,
```

```
PLAT933_ALERT_2_G Number of HKL-OMIT Records in Embedded .res File          3 Note
               -2  0  2,   0  0  2,   2  0  0,
PLAT941_ALERT_3_G Average HKL Measurement Multiplicity ..........         4.1 Low
PLAT955_ALERT_1_G Reported (CIF) and Actual (FCF) Lmax Differ by .          1 Units
PLAT969_ALERT_5_G The 'Henn et al.' R-Factor-gap value ..........       3.008 Note
              Predicted wR2: Based on SigI**2  2.98 or SHELX Weight  8.27
PLAT978_ALERT_2_G Number C-C Bonds with Positive Residual Density.          0 Info
```

```
   0 ALERT level A = Most likely a serious problem - resolve or explain
   3 ALERT level B = A potentially serious problem, consider carefully
  15 ALERT level C = Check. Ensure it is not caused by an omission or oversight
  20 ALERT level G = General information/check it is not something unexpected

   7 ALERT type 1 CIF construction/syntax error, inconsistent or missing data
  15 ALERT type 2 Indicator that the structure model may be wrong or deficient
   6 ALERT type 3 Indicator that the structure quality may be low
   3 ALERT type 4 Improvement, methodology, query or suggestion
   7 ALERT type 5 Informative message, check
```

It is advisable to attempt to resolve as many as possible of the alerts in all categories. Often the minor alerts point to easily fixed oversights, errors and omissions in your CIF or refinement strategy, so attention to these fine details can be worthwhile. In order to resolve some of the more serious problems it may be necessary to carry out additional measurements or structure refinements. However, the purpose of your study may justify the reported deviations and the more serious of these should normally be commented upon in the discussion or experimental section of a paper or in the "special_details" fields of the CIF. checkCIF was carefully designed to identify outliers and unusual parameters, but every test has its limitations and alerts that are not important in a particular case may appear. Conversely, the absence of alerts does not guarantee there are no aspects of the results needing attention. It is up to the individual to critically assess their own results and, if necessary, seek expert advice.

Publication of your CIF in IUCr journals

A basic structural check has been run on your CIF. These basic checks will be run on all CIFs submitted for publication in IUCr journals (*Acta Crystallographica*, *Journal of Applied Crystallography*, *Journal of Synchrotron Radiation*); however, if you intend to submit to *Acta Crystallographica Section C* or *E* or *IUCrData*, you should make sure that full publication checks are run on the final version of your CIF prior to submission.

Publication of your CIF in other journals

Please refer to the *Notes for Authors* of the relevant journal for any special instructions relating to CIF submission.

PLATON version of 22/08/2024; check.def file version of 21/08/2024

Datablock a - ellipsoid plot

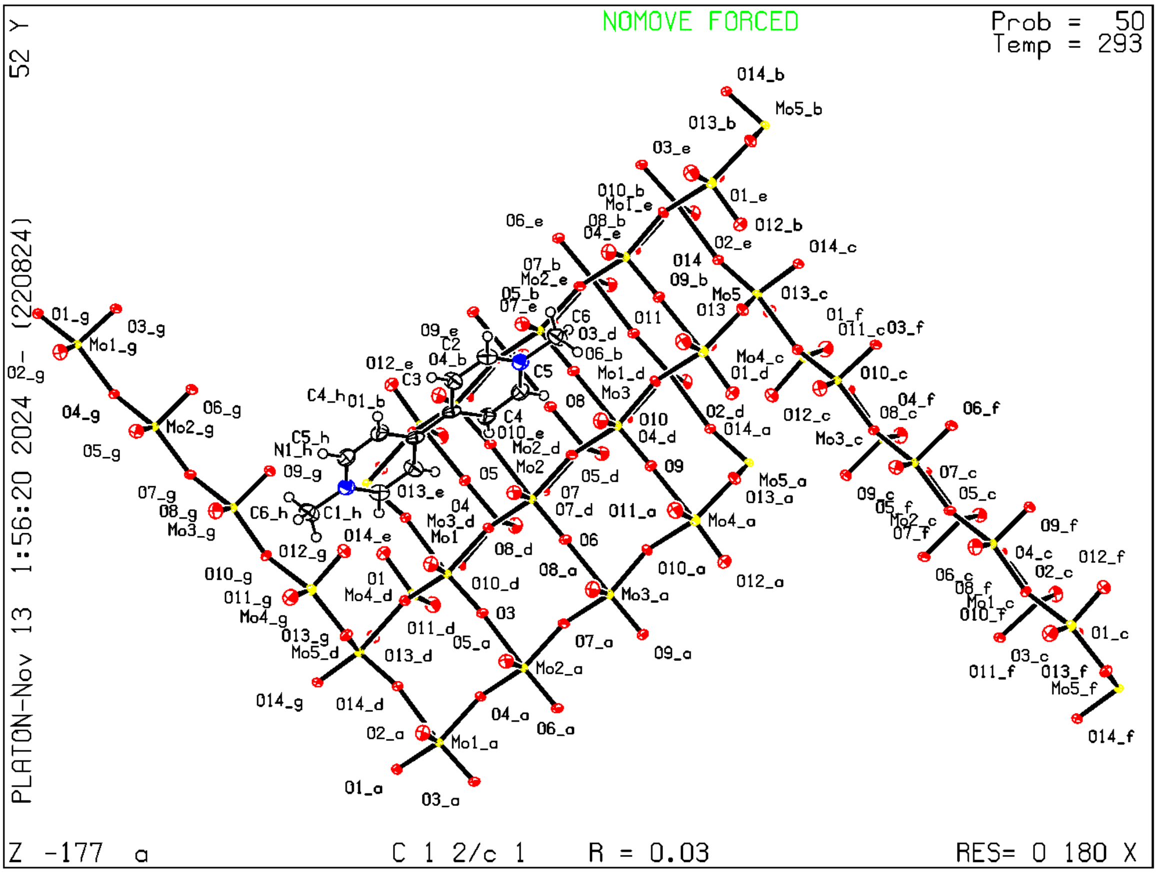

Supplement: Supplementary file 3 — Supporting Information [file ADVS-13-e19866-s001.zip › checkcif_compound 1.pdf]
